# Ancient Tethyan Vicariance and Long-Distance Dispersal Drive Global Diversification and Cryptic Speciation in the Red Seaweed *Pterocladiella*

**DOI:** 10.3389/fpls.2022.849476

**Published:** 2022-06-02

**Authors:** Ga Hun Boo, Frederik Leliaert, Line Le Gall, Eric Coppejans, Olivier De Clerck, Tu Van Nguyen, Claude E. Payri, Kathy Ann Miller, Hwan Su Yoon

**Affiliations:** ^1^Department of Biological Sciences, Sungkyunkwan University, Suwon, South Korea; ^2^Institut de Systématique, Evolution, Biodiversité (ISYEB), Muséum National d’Histoire Naturelle, CNRS, Sorbonne Université, EPHE, Paris, France; ^3^University Herbarium, University of California, Berkeley, CA, United States; ^4^Meise Botanic Garden, Meise, Belgium; ^5^Phycology Research Group, Department of Biology, Ghent University, Ghent, Belgium; ^6^Department of Ecology, Institute of Tropical Biology, Vietnam Academy of Science and Technology, Ho Chi Minh City, Vietnam; ^7^UMR Entropie (IRD, Ifremer, Univ Nouvelle-Calédonie, Univ La Réunion, CNRS), Nouméa, New Caledonia

**Keywords:** biogeography, Eastern Pacific Barrier, Gelidiales, molecular dating, overlooked biodiversity, sister species, Tethyan origin

## Abstract

We investigated the globally distributed red algal genus *Pterocladiella*, comprising 24 described species, many of which are economically important sources of agar and agarose. We used DNA-based species delimitation approaches, phylogenetic, and historical biogeographical analyses to uncover cryptic diversity and infer the drivers of biogeographic patterns. We delimited 43 species in *Pterocladiella*, of which 19 are undescribed. Our multigene time-calibrated phylogeny and ancestral area reconstruction indicated that *Pterocladiella* most likely originated during the Early Cretaceous in the Tethys Sea. Ancient Tethyan vicariance and long-distance dispersal have shaped current distribution patterns. The ancestor of Eastern Pacific species likely arose before the formation of the formidable Eastern Pacific Barrier—a first confirmation using molecular data in red algae. Divergences of Northeast and Southeast Pacific species have been driven by the Central American Seaway barrier, which, paradoxically, served as a dispersal pathway for Atlantic species. Both long- and short-distance dispersal scenarios are supported by genetic relationships within cosmopolitan species based on haplotype analysis. Asymmetrical distributions and the predominance of peripatry and sympatry between sister species suggest the importance of budding speciation in *Pterocladiella*. Our study highlights the underestimation of global diversity in these crucial components of coastal ecosystems and provides evidence for the complex evolution of current species distributions.

## Introduction

Seaweeds are essential components of coastal ecosystems worldwide, yet the lack of fossils, the absence of extensive, targeted collections, and the paucity of intensive molecular studies have limited our understanding of their historical biogeography. Seaweed biogeography initially focused on distribution patterns and physiological adaptations of species in relation to macroecological factors, particularly variation in temperature (e.g., Setchell, 1920; van den Hoek, 1982; Breeman, 1988; Bolton and Anderson, 1990; Lüning, 1990). More realistic estimations of species diversity using DNA-based species delimitation approaches in combination with advances in historical biogeographical modeling using phylogenetic frameworks has enabled the exploration of processes underlying biogeographical patterns (Verbruggen et al., 2007, 2009; Vieira et al., 2017, 2021; Leliaert et al., 2018; Yip et al., 2020). These studies have highlighted the need for a comprehensive understanding of species boundaries and geographic ranges, with well-resolved phylogenies based on taxon-wide and thorough geographic sampling, as a basis for probing evolutionary origins and historical distributions.

Recent global historical biogeographical studies of representative seaweeds have highlighted some commonalities and differences in patterns of species ranges, origin, and diversification. Benthic marine algae are typically genus rich in temperate seas (Kerswell, 2006), while species richness is high in the tropics, especially in the Central Indo-Pacific such as for the red seaweed *Portieria*, brown seaweed *Lobophora* and *Sargassum*, and green seaweed Udoteaceae (Vieira et al., 2017; Leliaert et al., 2018; Yip et al., 2020; Lagourgue et al., 2022). Although there are few genus-level studies, some are originated during the Cretaceous period from various regions, such as Australasia, Eastern Asia, or broad range in the Tethys Sea; their diversification occurred relatively constantly over time and long-distance dispersal event are more important mode of speciation than vicariance (Hommersand, 1990; Vieira et al., 2017; Leliaert et al., 2018; Xu et al., 2018).

The red algal genus *Pterocladiella* Santelices & Hommersand (Gelidiales, Rhodophyta) is globally distributed in tropical and temperate seas; the species are economically valuable because they provide agar for food and high-grade bacteriological and pharmaceutical agarose (Rioux and Turgeon, 2015; Santos and Melo, 2018). These species can be dominant components of coastal habitats and act as ecological engineers. Some species serve communities as nurseries for intertidal invertebrates, host diverse microbial communities, and provide food for marine grazers such as green turtles, fishes, gastropods, and sea urchins (Felicini and Perrone, 1994; Ibrahim et al., 2015; Campos and Cardona, 2020; Patarra et al., 2020). Although the genus, like most red algae, has a triphasic life cycle comprising tetrasporophytes, sexual gametophytes, and carposporophytes, male and female plants are rarely found in most species (Boo et al., 2010, 2017; Iha et al., 2017; Patarra et al., 2020). Thus, reproduction likely depends on asexual, non-motile tetraspores or the regeneration of vegetative fragments or holdfasts, and the capacity of regeneration may help proliferate during sporadic dispersal events.

*Pterocladiella* was established on the basis of four species, previously placed in the genus *Pterocladia* J.Agardh; the generitype is *P. capillacea* (S.G.Gmelin) Santelices & Hommersand (Santelices and Hommersand, 1997). To date, 24 species have been described, many using morphological characters in combination with molecular markers (Shimada et al., 2000; Thomas and Freshwater, 2001; Tronchin and Freshwater, 2007; Freshwater et al., 2010; Sohrabipour et al., 2013; Boo and Geraldino, 2016; Boo et al., 2016a, 2016b, 2017; Iha et al., 2017; Wang et al., 2020). Boo et al. (2017) found that *P. caloglossoides* (M.Howe) Santelices from Peru, the type locality, was distantly related to the Australian taxa identified by Millar and Freshwater (2005), indicating that specimens from other regions (e.g., China, Hawai’i, etc.; Guiry and Guiry, 2022) may also belong to different species. In contrast, *P. capillacea* has been reported to occur globally (Freshwater et al., 1995; Shimada et al., 2000; Boo et al., 2010; Iha et al., 2017; Wang et al., 2020). These studies have raised a number of important questions at the heart of our study of the global and temporal diversification of *Pterocladiella*: are allegedly widespread species actually composed of multiple species with narrow ranges, and do broadly distributed species maintain genetic connectivity over global distances? Where and how did these species arise and disperse on an evolutionary time scale?

The goal of this study is to gain a comprehensive picture of the global diversification of *Pterocladiella* by (i) evaluating species diversity using DNA-based species delimitation analyses of taxon-wide samples and (ii) building a multigene time-calibrated phylogeny and estimating ancestral ranges using molecular dating and historical biogeographical analysis. Our is the first study to address these issues in a globally distributed red algal genus, and we discuss potential modes of speciation and compare our results with recent studies of spatial and temporal patterns of diversification in other seaweeds, such as *Lobophora*, *Sargassum*, and *Portieria* (Vieira et al., 2017; Leliaert et al., 2018; Yip et al., 2020).

## Materials and Methods

### Taxon Sampling

**Figure 1:**
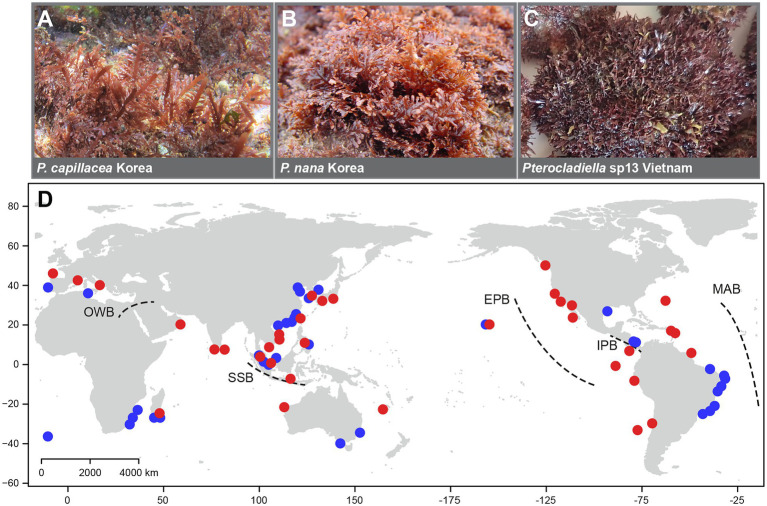
Morphological diversity in the genus **(A–C)** and map **(D)** of collection sites of *Pterocladiella* specimens with five major marine biogeographic barriers: the East Pacific Barrier (EPB), the Isthmus of Panama Barrier (IPB), the Old World Barrier (OWB), the Mid-Atlantic Barrier (MAB), and the Sunda Shelf Barrier (SSB; Rocha et al., 2007; Toonen et al., 2016). Red circles indicate newly collected sites and blue circles indicate previously analyzed sites.

Samples were collected worldwide and included 92% (22) of the 24 described species, covering most of the geographical range of the genus *Pterocladiella* (Figure 1). Samples were identified in the field or with microscopy in the laboratory and confirmed by DNA sequencing (see below). They were air-dried or preserved in silica gel for molecular analysis. We obtained apical fragments approximately 5 mm in size from type and a selection of archival herbarium specimens, with the permission of curators at the cryptogam herbarium of the Muséum National d’Histoire Naturelle in Paris, France (PC), the Ghent University macroalgal herbarium, Ghent, Belgium (GENT; collection now housed in the herbarium of Meise Botanic Garden, BR), the herbarium of Naturalis Biodiversity Center in Leiden, Netherlands (L), and the University Herbarium, University of California at Berkeley, United States (UC; herbarium abbreviations follow Thiers, 2021). In total, 319 specimens from 36 countries, including 157 newly analyzed specimens, were included in this study (Supplementary Table 1).

### DNA Extraction and Multi-Locus Sequencing

DNA extraction, PCR amplification, and sequencing were followed by Boo et al. (2016a,c). Five markers, known to be phylogenetically informative in the Gelidiales (Freshwater and Rueness, 1994; Freshwater et al., 2010; Boo et al., 2016c, 2020), were selected for molecular analyses: two mitochondrial (COI-5P and *cob*) and three plastid (*psa*A, *psb*A, and *rbc*L) markers. Primer sequences for the five markers are provided in Supplementary Table 2. We first sequenced COI-5P from fresh and archival herbarium specimens and then sequenced *cob*, *psa*A, *psb*A, and *rbc*L to construct our species phylogeny. Despite degradation or fragmentation of DNA from the archival specimens (mostly <200 bp; Hughey et al., 2014), we were able to amplify and sequence 245–584 bp of *rbc*L using primers in Supplementary Table 2. Sequencing was performed by Genotech Co. (Daejeon, Korea) and Bioneer (Daejeon, Korea). Newly generated 299 sequences were deposited in GenBank: 135 COI-5P, 40 *cob*, 36 *psa*A, 41 *psa*A, and 47 *rbc*L sequences (Supplementary Table 1). Sequences were aligned using the MUSCLE algorithm in MEGA7 (Kumar et al., 2016) with default parameters, and the alignments were manually adjusted. The datasets, including previously published sequences of *Pterocladiella* from GenBank, contained 307 COI-5P, 45 *cob*, 43 *psa*A, 48 *psb*A, and 140 *rbc*L sequences.

### Species Delimitation Analysis

We assembled two datasets with the most extensive taxon sampling: mitochondrial COI-5P and plastid *rbc*L. The alignment of 307 COI-5P and 140 *rbc*L sequences was first reduced to 132 and 81 unique haplotypes, respectively. Only *rbc*L data were available for *P. bulbosa*, *P. caespitosa*, *Pterocladiella* sp.16 (as *P. caloglossoides* from Australia; Millar and Freshwater, 2005), and “*Gelidiella feldmannii*.” Modeltest v.3.7 (Posada and Crandall, 1998) using the Akaike Information Criterion (AIC) identified the GTR + G + I as the best fit model for both datasets. Ultrametric trees were obtained by Bayesian analyses in BEAST v1.10.4 (Suchard et al., 2018), with divergence times estimated under an uncorrelated lognormal relaxed molecular clock model (Drummond et al., 2012) and the Yule-Process as the tree prior. Markov Chain Monte Carlo (MCMC) analyses were run separately four times for 50 million generations, with subsampling every 5,000 generations. The output was checked for convergence using Tracer v.1.7 (Rambaut et al., 2018). The maximum clade credibility (MCC) tree was generated after discarding 25% of the saved trees with TreeAnnotator v1.10.4.

Four species delimitation methods were applied. The single- and multiple-threshold generalized mixed Yule coalescent methods (GMYC; Pons et al., 2006; Fujisawa and Barraclough, 2013) were performed on the MCC tree using the SPLITS package in R 3.5.2 (R Core Team, 2018). The Bayesian implementation of the Poisson tree processes method (bPTP; Zhang et al., 2013) was conducted through the web server,^1^ with the following settings: 500,000 MCMC generations, thinning by a factor of 100, and 10% burn-in. The automatic barcode gap discovery analysis (ABGD; Puillandre et al., 2012) was conducted through the webserver^2^ under the default parameters, except related gap width (X) as 1.3, using Kimura-2-parameter (K2P) distance matrix as input file, generated in MEGA7. However, because of a number of partial *rbc*L sequences (245–584 bp), ABGD analysis of the *rbc*L dataset was not possible.

### Phylogenetic Analyses

Our species phylogeny was based on five markers and our delimited species. On the basis of the previous phylogenetic studies of the Gelidiales (Freshwater et al., 1995; Boo et al., 2016c), six species in the Gelidiales were selected as outgroups (Supplementary Table 1). Phylogenies of individual and concatenated datasets were reconstructed using Maximum Likelihood (ML) and Bayesian inference (BI). Modeltest v.3.7 using the AIC identified the GTR + G + I as the best fit model for each of the five datasets. For our five-gene concatenated dataset (4,485 bp), PartitionFinder v.2.1.1 (Lanfear et al., 2017) was used to determine the best-fit partitioning schemes and models of molecular evolution using the greedy algorithm with unlinked branch lengths. The concatenated alignment was divided in two partitions, each with a GTR + G + I model: (i) mitochondrial COI-5P + *cob* and (ii) plastid *psa*A + *psb*A + *rbc*L. The ML analyses were performed using the W-IQ-tree webserver (Trifinopoulos et al., 2016) with 1,000 ultrafast bootstrap (BS) replicates. The BI was performed with MrBayes v.3.2.1 (Ronquist et al., 2012) using the Metropolis-coupled Markov Chain Monte Carlo (MC3) with the models and partitions selected by Modeltest and PartitionFinder. Four million generations of two independent runs were performed with four chains and sampling trees every 100 generations. The burn-in period was identified graphically by tracking the likelihoods at each generation to determine when they reached a plateau. Twenty-five percent of saved trees were removed, and the remaining trees were used to calculate the Bayesian posterior probabilities (BPPs).

### Multilocus Time-Calibrated Phylogeny

The age of the root of the genus *Pterocladiella* was estimated with BEAST using an uncorrelated lognormal relaxed molecular clock model and the Yule-Process as the tree prior. For this analysis, a four-gene dataset (COI-5P, *psa*A, *psb*A, and *rbc*L sequences), including representatives of the Gelidiales and five *Pterocladiella* lineages recognized in this study, was assembled as described by Yang et al. (2016). Mitochondrial *cob* was excluded because it was not used in Yang et al. (2016). DNA sequences were aligned for each marker separately using the MUSCLE algorithm in MEGA7 with amino acid translations taken into account for protein coding regions. The four alignments were then concatenated into a single alignment of 1,472 positions, which was 91% filled at the species × locus level. A suitable partitioning scheme and accompanying substitution model was selected using PartitionFinder with the Bayesian information criterion (BIC). The BIC identified the CPREV + G + I model as one partition. Since fossils are not available to serve as internal calibration points within the Gelidiales (Boo et al., 2018), we applied secondary age constraints, including standard deviations (SD), derived from Yang et al. (2016). Three calibration points were used with a normal prior distribution: (1) the age of the Rhodymeniophycidae (*M* = 412 Ma, SD = 30), (2) the split between the Ceramiales and Acrosymphytales (*M* = 335 Ma, SD = 28), and (3) the crown node of Rhodymeniales + Sebdeniales + Halymeniales + Nemastomatales + Gracilariales + Plocamiales + Gelidiales (*M* = 340 Ma, SD = 30). Four independent MCMC analyses of 50 million generations were performed, sampling every 5,000 generations. The root age of *Pterocladiella* was estimated at 128 Ma (171–89 Ma), which was used as a constraint in further estimating the age of *Pterocladiella* species.

A time-calibrated phylogeny of *Pterocladiella* species was constructed with BEAST based on the concatenated dataset (COI-5P, *cob*, *psa*A, *psb*A, and *rbc*L sequences). PartitionFinder, according to the BIC, identified two partitioning schemes (mitochondrial and plastid markers) with the GTR + I + G model for each partition. Data were analyzed using a Yule-Process tree prior, an uncorrelated log normal relaxed clock model of rate variation among branches. The root of the tree (*Pterocladiella*) was constrained with a normal prior distribution (*M* = 128 Ma, SD = 21). Four independent MCMC analyses of 30 million generations were performed, sampling every 3,000 generations, to obtain posterior distributions of parameters excluding a burn-in of 10%. Convergence of each analysis was determined in Tracer, examining the effective sampling size (ESS) for all parameters. For the analysis using two data partitions, the ESS was >200 for all parameters. The MCC tree was generated with TreeAnnotator after discarding 10% of the saved trees as burn-in.

### Inference of Biogeographic History and Diversification Rates

Ancestral ranges were estimated based on the time-calibrated phylogeny using BioGeoBEARS (Matzke, 2013a) in RASP v.4.2 (Yu et al., 2020), an R package implementing several ancestral range estimation models in a likelihood framework. We analyzed our data under three models, Dispersal-Extinction Cladogenesis (DEC; Ree and Smith, 2008), a likelihood version of the parsimony-based Dispersal-Vicariance Analysis (DIVALIKE; Ronquist, 1997), and a likelihood version of the range evolution model implemented in BayArea (BAYAREALIKE; Landis et al., 2013). These models allow for a wide range of processes, including within-area speciation, vicariance, range expansion and extinction (Matzke, 2013b). DEC assumes that daughter lineages inherit the ancestral range if the ancestor lives in a single area, or if the ancestor is widespread, one daughter lineage will live in a subset of this area, or one area will split off by vicariance. On the other hand, DIVALIKE allows this form of vicariance but disallows subset speciation; BAYAREALIKE assumes that no range evolution occurs at cladogenesis (Matzke, 2013b). Three models were compared for statistical fit using the corrected Akaike Information Criterion (AICc).

Two biogeographical subdivisions were based on current species distributions. First, three regions were considered: Indo-West Pacific, Eastern Pacific, and Atlantic. Second, we considered eight realms modified from Spalding et al. (2007): (A) Central Indo-Pacific, (B) Western Indo-Pacific, (C) Eastern Indo-Pacific, (D) Northwestern Pacific, (E) Temperate Australasia, (F) Eastern Pacific, (G) Western Atlantic, and (H) Eastern Atlantic, including the Mediterranean Sea. Geographical distributions were based on location data of the 319 sequenced specimens. For *P. capillacea*, based on our phylogeny, the recent range-expanded areas were excluded from the species’ geography matrix; thus, the area of *P. capillacea* was adjusted here to the Northwestern Pacific. The maximum number of areas for a single species was set at three for the analyses.

Shifts in diversification rate through time and among lineages were tested using Bayesian analysis of macroevolutionary mixtures (BAMM; Rabosky, 2014). The BEAST tree was used as input with expected number of shifts = 1, 20 million generations of MCMC sampling per run, and sampling evolutionary parameters every 2,000 generations. A lineage-through-time (LTT) plot, including a 95% CI based on a set of 1,000 post burn-in trees, was generated using Phytools (Revell, 2012).

### Genetic Structure of Cosmopolitan Species Using COI-5P Haplotypes

To analyze relationships and long-distance dispersal within three cosmopolitan species occurring in at least three biogeographical realms, we constructed haplotype networks. Haplotype networks of COI-5P sequences were built using the TCS method (Clement et al., 2000) with Popart 1.7 (Leigh and Bryant, 2015). However, due to the sampling of an uneven number of populations, we did not analyze population structure using other statistical methods.

## Results

### Species Delimitation, Diversity, and Geographical Distribution

**Figure 2:**
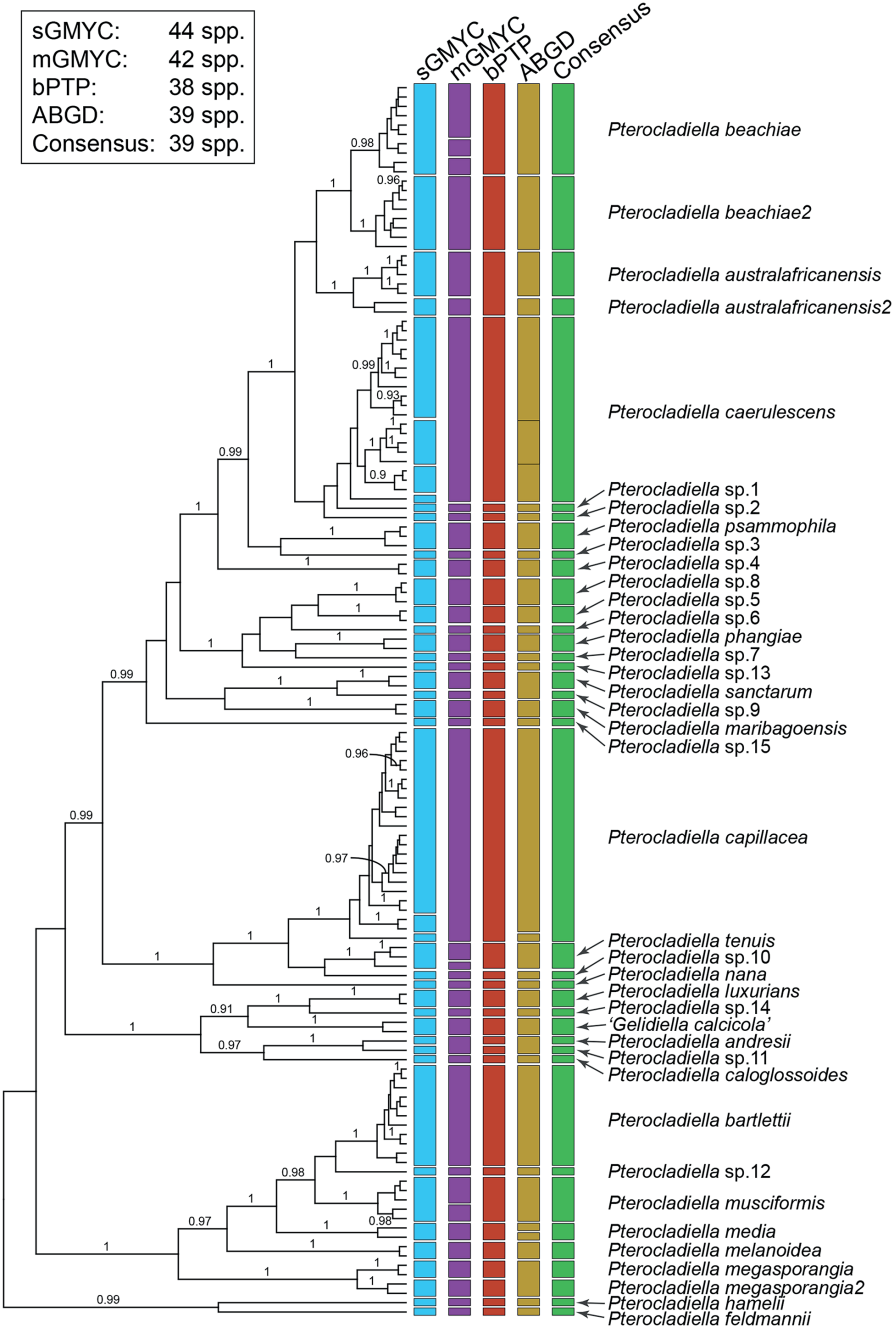
Results of the four species delimitation methods based on the *cox*1 dataset. Bars represent results from the generalized mixed Yule coalescent (GMYC) method using a single threshold (sGMYC), GMYC with multiple thresholds (mGMYC), Bayesian poison tree processes method (bPTP), and automatic barcode gap discovery (ABGD). The green bar represents the consensus species delimitation using a conservative approach that matched at least three out of four species delimitation analyses. Posterior probability is indicated on the nodes.

Results of the DNA-based species delimitation analyses for mitochondrial COI-5P and plastid *rbc*L sequences are given in Figure 2; Supplementary Figure 1; Supplementary Table 3. For COI-5P, the single and multiple threshold models of GMYC (sGMYC and mGMYC), bPTP, and ABGD indicated 38–44 putative species (Figure 2). We recognized 39 species, including “*Gelidiella calcicola*,” by selecting groups that were uncovered by at least three out of the four species delimitation analyses. *Pterocladiella australafricanensis*, *P. beachiae*, and *P. megasporangia* were each found to consist of pairs of cryptic sister species. *Pterocladiella australafricanensis* was divided into two species in the sGMYC, mGMYC, and ABGD analyses; *P. australafricanensis* shared the sequence of the type specimen from South Africa and also occurred in Brazil and Mozambique, while *P. australafricanensis2* included specimens from Madagascar and Oman. In the sGMYC, bPTP, and ABGD analyses, *P. beachiae* shared the type sequence from Costa Rica, and a second species, *P. beachiae2*, was collected in the Central Indo-Pacific. In the sGMYC, mGMYC, and bPTP analyses, *P. megasporangia* also comprised two species; *P. megasporangia* included the type sequence from Malaysia, and the other species, *P. megasporangia2*, was represented by specimens from Indonesia, Taiwan, and Vietnam.

The species delimitation analyses of *rbc*L sequences were largely congruent with those of COI-5P. Four species analyzed only with *rbc*L sequences were distinct from the others. A comparison of a partial *rbc*L sequence (245 bp) from the holotype of *P. bulbosa* (UC1884014 in UC) generated in this study, and three published sequences from *P. caespitosa* from South Africa, *Pterocladiella* sp16 from Australia, and “*Gelidiella feldmannii*” from Tristan da Cunha, showed that these species are indeed distinct (Supplementary Figure 1). Because the *cox*1 marker is highly informative for very recent divergence, while the phylogenetic informativeness of *rbc*L is useful at the genus- or family-level in the Gelidiales (Boo et al., 2016c), delimiting *Pterocladiella* species using a single marker should be used cautiously, and with comprehensive taxon sampling.

Our final species delimitation analyses, based on the COI-5P consensus result complemented with the *rbc*L analysis, included 43 species in which four species were delimited by *rbc*L sequences. The geographical distributions of the 43 *Pterocladiella* species, based on specimens for which sequence data are available, are summarized in Supplementary Table 4. Thirty-five species are restricted to a single realm (endemic), while eight species are widespread. Three of these occurred in more than three realms (i.e., cosmopolitan: *P. bartlettii*, *P. caerulescens*, and *P. capillacea*), and five species occurred in two realms (i.e., subcosmopolitan: *P. australafricanensis*, “*P. beachiae2*,” *P. media*, *P. musciformis*, and *Pterocladiella* sp3; Supplementary Table 4).

The highest species diversity was found in the Central Indo-Pacific (18 spp. from 142 specimens), followed by the Western Indo-Pacific (11 spp. from 20 specimens), the Eastern Pacific (eight spp. from 16 specimens), and the Western Atlantic (eight spp. from 96 specimens). Species diversity was lower (2–4 spp.) in the Eastern Indo-Pacific (six specimens), Northwestern Pacific (26 specimens), Temperate Australasia (two specimens), and Eastern Atlantic (11 specimens).

The latitudinal range 0–20°N contained the largest number of species (23 spp.), followed by 21°N–40°N (18 spp.) and 21°S–40°S (12 spp.). Smaller numbers of species were present in 0–20°S (five spp.), and 41°N–60°N (three spp.), and none was present in 41°S–60°S (Supplementary Table 5). Latitudinal and longitudinal range sizes of species are illustrated in Supplementary Figure 2.

### Phylogenetic Reconstruction

Our species phylogeny, comprising 43 *Pterocladiella* species and six outgroups based on the five-gene (COI-5P, *cob*, *psa*A, *psb*A, and *rbc*L sequences; 4,458 bp) concatenated alignment, was generally concordant with the individual gene phylogenies (Supplementary Figures 3, 4), but node support was considerably higher in the concatenated dataset. The ML and BI analyses revealed mostly congruent topologies; the ML tree with branch supports (BS and BPP) is shown in Supplementary Figure 3. *Pterocladiella* was monophyletic (BS: 100, BPP: 1.0) and consisted of six main clades (I–VI), with most of the backbone nodes well supported.

Clade I (BS: 100, BPP: 1.0) was the largest, including 20 species from the Indo-Pacific and the Western Atlantic. Clade II (BS: 100, BPP: 1.0) included *P. nana*, *P. tenuis*, and *Pterocladiella* sp10 from East Asia, plus a cosmopolitan species, *P. capillacea*. Clade III consisted of Australian *Pterocladiella* sp16. Clade IV (BS: 100, BPP: 1.0) comprised seven species from the Eastern Pacific and two species from the Atlantic Ocean. Clade V (BS: 100, BPP: 1.0) consisted of four widespread species (*P. bartlettii*, *P. media*, *P. megasporangia2*, and *P. musciformis*) and four species from the Central Indo-Pacific, the Eastern Indo-Pacific, and the Eastern Atlantic. Clade VI, including *P. caespitosa*, *P. feldmannii*, and *P. hamelii* from Madagascar or South Africa, was consistently separated from, and basal to, the rest of the *Pterocladiella* species.

### Divergence Time Estimates and Historical Biogeography

The time-calibrated phylogenetic analysis estimated that the root of the genus *Pterocladiella* was 128.4 Ma [95% highest posterior density (HPD): 171.2–89.4 Ma], indicating an Early Cretaceous origin of the genus (Table 1; Supplementary Figures 5, 6). The rate of diversification within the genus was relatively constant over time, with neither the LTT plot nor the BAMM analysis showing evidence for rate shifts (Supplementary Figure 7).

**Table 1:** Divergence time estimates and ancestral area of deep nodes in *Pterocladiella* inferred from BEAST and DIVALIKE model provided by BeoGeoBEARS.

Node and description	Mean divergence time (95% HPD; Ma)	BS/BPP	Ancestral area (most probable area)	
			Analyzed for three regions	Analyzed for eight realms
Root, genus *Pterocladiella*	111.7 (157.0–68.3)	100/1.0	Indo-Western Pacific	Western and Central Indo-Pacific
Node a, Groups 1–5	103.9 (144.2–60.3)	100/1.0	Indo-Western Pacific	Central Indo-Pacific
Node b, Groups 1–4	87.8 (123.6–52.5)	100/1.0	Indo-Western Pacific and Eastern Pacific	Central Indo-Pacific and Eastern Pacific
Node c, Groups 1–3	68.9 (99.6–40.6)	99/1.0	Indo-Western Pacific	Central Indo-Pacific, Northwestern Pacific, and Temperate Australasia
Node d, Group 5	63.5 (91.7–35.7)	100/1.0	Indo-Western Pacific and Atlantic	Central Indo-Pacific and Eastern Atlantic
Node e, Group 1	51.7 (75.4–30.6)	100/1.0	Indo-Western Pacific	Western and Central Indo-Pacific
Node f, Group 4	42.5 (61.7–23.9)	100/1.0	Eastern Pacific	Eastern Pacific
Node g, Group 2	30.6 (45.6–15.5)	100/1.0	Indo-Western Pacific	Northwestern Pacific

Node letters are same in the Supplementary Figure 5. Detailed geographical scales are explained in section Material and Methods. HPD, highest posterior density; BS, bootstrap support value; and BPP, Bayesian posterior probability.

**Figure 3:**
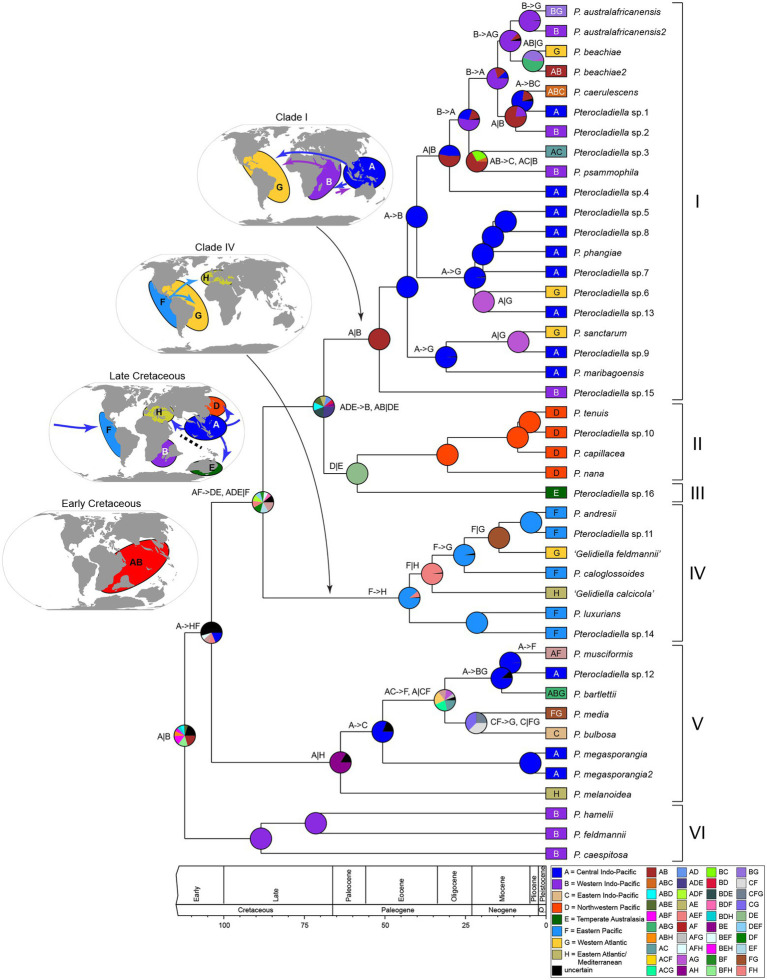
Ancestral area reconstructions and biogeographical events in *Pterocladiella* under DIVALIKE model considering eight geographic realms. Boxes at the tips indicate geographical ranges of extant *Pterocladiella* species. Pie charts represent the probabilities for the ancestral area of nodes. The map shows eight realms used in the analysis. A color key is provided in the figure. The inferred biogeographic events are indicated at the nodes and branches (e.g., A- > B: dispersal and A|B: vicariance).

The biogeographic model DIVALIKE was favored on the basis of AICc weights at both the level of the three regions and eight realms analyses (Supplementary Table 6). The inferred realm-level biogeographical history is shown in Figure 3; the region-level biogeographical history is shown in Supplementary Figure 8. In the description of the results below, we focused on the statistically well-supported phylogenetic relationships.

The six main clades were mostly confined to a single or a few adjacent regions or realms. In the region-level analysis, the Indo-West Pacific was inferred as the ancestral range of *Pterocladiella* (Supplementary Figure 7). In the realm-level analysis, the ancestral range was inferred as the Western and Central Indo-Pacific (AB; Figure 3). The most recent common ancestor (MRCA) of *Pterocladiella* was widely distributed in the Tethys Sea of the Early Cretaceous.

Throughout evolutionary history, both dispersal and vicariant events shaped current distribution patterns of *Pterocladiella*. The MRCA of the genus was first segregated by a vicariant event separating the Western Indo-Pacific and the Central Indo-Pacific. The analyses estimated several long-distance dispersal events in the early evolution of the genus to Northwestern Pacific, Temperate Australia, Eastern Pacific, and Eastern Atlantic during the Cretaceous period. The MRCA of clade I probably arose in the Western and Central Indo-Pacific during the Eocene (51.7 Ma, 95% HDP: 75.4–30.6 Ma), with most (60%) of the species maintaining a Central Indo-Pacific distribution. Two dispersal events from the Central Indo-Pacific to the Western Atlantic, followed by vicariance events, account for the current distribution of *P. sanctarum* and *Pterocladiella* sp.6. Similarly, two dispersal events from the Western Indo-Pacific to the Western Atlantic were inferred, resulting in cryptic sister species complexes for *P. australafricanensis* and *P. beachiae*, likely *via* the Mediterranean Sea before closure of the OWB barrier (c.a. 13 Mya). Clade II was estimated to have originated in the Northwestern Pacific during the Oligocene (30.6 Ma, 95% HDP: 45.6–15.6 Ma).

Clade IV was estimated to have originated in the Eastern Pacific during the Eocene (42.5 Ma, 95% HDP: 61.7–23.9 Ma). After long-distance dispersal from the Central Indo-Pacific, species diversified along the Pacific coast of America. Vicariance events between the Eastern and Western Atlantic likely resulted in the origin of “*G. calcicola*” and “*G. feldmannii*.” Clade V was estimated to arise in the Central Indo-Pacific and the Eastern Atlantic *via* a vicariance event during the Paleocene (63.5 Ma, 95% HDP: 91.7–35.7 Ma). Subsequently, several dispersal events to the Eastern Indo-Pacific, the Eastern Pacific, and the Western Atlantic account for current distributions in this clade.

Of 43 species, nine pairs of sister species, strongly supported in BS and BPP, revealed three speciation patterns: allopatry, peripatry, and sympatry. Drivers of divergence in relation to distribution (divergence time, sea surface temperature, distribution, and the shortest distance between species in these pairs) are summarized in Table 2.

**Table 2:** Divergence time and distribution of statistically supported (80 ≥ BS and 0.9 ≥ BPP) sister species in *Pterocladiella*.

Distribution (allo- peri-, sympatry) Species pair	BS/BPP	COI-5P divergences (%)	Divergence time (95% HPD), Ma	Average sea surface temperature	Occurrence
Allopatry *Pterocladiella sanctarum* *Pterocladiella* sp.9	100/1.0	3.6–3.8	8.5 (14.9–3.3)	26.8–28.9°C vs. 26.4–29.1°C	Guadeloupe/Philippines
Allopatry *P. beachiae* *P. beachiae2*	100/1.0	1.7–3.8	3.9 (6.6–1.5)	27.3–29.1°C vs. 29.1–30.6°C	Brazil, Costa Rica, Guadeloupe, Martinique, Panama/China, India, Malaysia, and Thailand
Peripatry *P. luxurians* *Pterocladiella* sp.14	100/1.0	7.9	21.5 (33.1–10.9)	15.0–21.1°C vs. 6.6–18.7°C	United States (southern California)/United States (Monterey), and Canada (British Columbia)
Peripatry *P. andresii* *Pterocladiella* sp.12	100/1.0	2.9	4.7 (7.6–2.0)	12.9–17.8°C vs. 14.1–19.8°C	Chile (Coquimbo)/Chile (Robinson Crusoe Island)
Peripatry *P. megasporangia* *P. megasporangia2*	100/1.0	1.8–2.5	4.8 (8.1–1.8)	28.5–30.4°C vs. 28.2–30.7°C	Malaysia/Indonesia, Taiwan, and Vietnam
Peripatry *P. australafricanensis* *P. australafricanensis2*	100/1.0	2.3–3.1	5.0 (8.3–2.0)	22.4–27.4°C vs. 24.0–29.2°C	Brazil, Mozambique, South Africa/Madagascar, and Oman
Sympatry *Pterocladiella* sp.5 *Pterocladiella* sp.8	89/0.9	8.4–8.8	12.5 (18.9–6.4)	28.2–30.7°C	Vietnam/Vietnam
Sympatry (partial) *P. caerulescens* *Pterocladiella* sp.1	96/1.0	3.8–5.0	7.3 (11.4–3.6)	23.7–28.1°C	Australia (Western Australia), China, UA (Hawai’i), Madagascar, Malaysia, New Caledonia, Philippines, Singapore, Sri Lanka, and Vietnam/New Caledonia
Sympatry *P. tenuis* *Pterocladiella* sp.10	100/1.0	3.7–4.6	4.9 (7.9–2.2)	8.0–25.6	Korea and Japan/Korea

The average sea surface temperature is based on https://seatemperature.info.

### Haplotype Networks of Cosmopolitan Species

**Figure 4:**
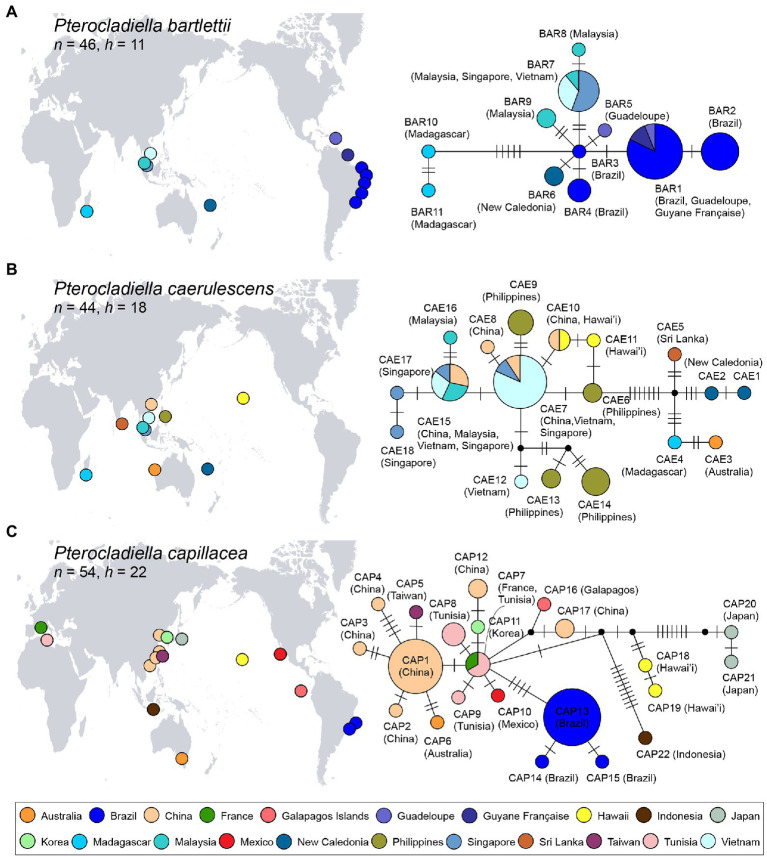
Map of the distributions and haplotype networks of three cosmopolitan species, *Pterocladiella bartlettii* **(A)**, *P. caerulescens* **(B)**, and *P. capillacea* **(C)** based on mitochondrial COI-5P sequences. Haplotypes are colored according to country as shown in the map. Each circle denotes a single haplotype with size proportional to frequency. *n*, number of specimens and *h*, number of haplotypes.

For three cosmopolitan species, statistical parsimony networks of COI-5P haplotypes are shown in Figure 4. *Pterocladiella bartlettii* was represented by 11 haplotypes from 46 specimens, with most of them directly connected with the haplotype BAR3 from Brazil (Figure 4A). A single haplotype BAR1 was widespread in the Western Atlantic, while BAR7 occurred in Southeast Asia. Southeast Asian haplotypes were relatively closely related to the Western Atlantic haplotypes including New Caledonia (3–5 mutations), while the Madagascar haplotypes, separated by 6–9 mutations, were distantly related.

*Pterocladiella caerulescens* comprised 18 haplotypes from 44 specimens (Figure 4B). Hawaiian haplotypes were closely related to those from Southeast Asia. Haplotype CAE10 was found in both China and Hawai’i. Haplotypes from Sri Lanka, New Caledonia, Madagascar and Western Australia (CAE1–CAE5) were distantly connected to those from Southeast Asia and Hawai’i.

The *Pterocladiella capillacea* network revealed multiple clusters, comprising 22 haplotypes from 53 specimens (Figure 4C). Haplotype CAP7 from France and Tunisia was closely related to haplotypes from China, Korea, the Galápagos Islands, Ecuador, and Mexico by two mutation steps, respectively. However, haplotypes CAP13–CAP15 from Brazil were five to seven mutation steps from CAP7. Haplotypes from Hawai’i (CAP18–CAP19), Japan (CAP20–CAP21), and Indonesia (CAP22) were segregated by many missing haplotypes.

## Discussion

### High Species Diversity

The species diversity in *Pterocladiella*, estimated in this study using DNA-based species delimitation methods, is remarkably high with 43 species including 19 undescribed and two species erroneously placed in *Gelidiella*. Our intensive and spatially comprehensive collection of specimens, including many that are tiny and inconspicuous, was critical to the discovery of these cryptic or overlooked species. These results are particularly notable because they nearly double the species number in *Pterocladiella*, despite numerous previous taxonomic studies (Shimada et al., 2000; Santelices, 2007; Tronchin and Freshwater, 2007; Freshwater et al., 2010; Sohrabipour et al., 2013; Boo et al., 2016a, 2016b, 2017; Iha et al., 2017; Wang et al., 2020). Simple morphology and rare occurrence of sexual reproductive structures (Boo et al., 2010; Patarra et al., 2020), both of which reduce the number of morphological characters, plus a high degree of phenotypic plasticity, have hindered the discriminating power of traditional taxonomy of *Pterocladiella*. *Pterocladiella* can be added to the list of red algal taxa for which DNA sequences have resulted in a striking increase in species diversity, such as *Portieria*, *Polysiphonia*, Bangiales, and coralline red algae known as rhodoliths or maerl (Payo et al., 2013; Pardo et al., 2014; Guillemin et al., 2016; Leliaert et al., 2018; Pezzolesi et al., 2019; Díaz-Tapia et al., 2020; Sissini et al., 2021).

To anchor the delimited species to published names, we included DNA sequences of the holotype of *P. bulbosa*, and topotype materials of *P. caerulescens* and *P. sanctarum* (Supplementary Tables 1, 4). We reliably associated type or topotype sequences to 22 of the 24 currently accepted species names in the genus. We also found that *Gelidiella calcicola* Maggs & Guiry and *G. feldmannii* Baardseth fall within the genus *Pterocladiella*. *Gelidiella calcicola* typically grows on subtidal maerl in northern France and England (Maggs and Guiry, 1987). However, Díaz-Tapia and Bárbara (2014) remarked that intertidal *P. melanoidea* from the Atlantic Iberian Peninsula largely resembled *G. calcicola*. Our collections from both intertidal rock and subtidal maerl in northern France match *P. melanoidea sensu* Díaz-Tapia and Bárbara (2014) in both COI-5P sequence and morphology (figure not shown), but they are distantly related to specimens identified as *P. melanoidea* in the Mediterranean Sea (Figure 2; Supplementary Figure 3). We conclude that our taxon is the same as *Gelidiella calcicola*. *Gelidiella feldmannii* is a species described from Tristan da Cunha (Baardseth, 1941). Saunders et al. (2019) suggested that it likely belongs in *Pterocladiella* on the basis of *rbc*L sequence analysis. After further morphological observations (especially of reproductive structures), we will be able to describe and name the undescribed taxa in this study.

Some regions remain under-sampled in this study, including South Pacific islands and the Red Sea, where *Pterocladiella* has been reported (e.g., N’Yeurt and Payri, 2010; Einav et al., 2021). A denser sampling effort may uncover more undescribed species in these regions.

### Center of Species Diversity

Species richness was found to be highest in the Central Indo-Pacific (18 spp.). This region, part of the inferred ancestral range of *Pterocladiella*, is also known as a center of diversity for a wide range of tropical marine animals (Renema et al., 2008; Bowen et al., 2013; Cowman et al., 2017), as well as seaweeds (Vieira et al., 2017, 2021; Leliaert et al., 2018; Yip et al., 2020). Other areas of high species richness are the Western Indo-Pacific (11 spp.), the Eastern Pacific (eight spp.), and the Western Atlantic (eight spp.).

The proportion of *Pterocladiella* species occurring in a single realm is 81% (35 species; Supplementary Table 4). This high proportion of endemic species is similar to that in other seaweeds, such as *Portieria* (88%) and *Lobophora* (75%; Vieira et al., 2017; Leliaert et al., 2018). High levels of species diversity and endemism in the Central Indo-Pacific may be interpreted by both center of origin and center of accumulation models. Our results support studies of coral reef fishes, gastropods, and seaweeds that have suggested that the Indo-Malay biodiversity hotspot resulted from a combination of speciation within the region and species accumulation *via* dispersal (Barber and Bellwood, 2005; Williams and Duda, 2008; Vieira et al., 2017; Leliaert et al., 2018).

### Cretaceous Origin and Global Diversifications

A firm account of species richness and distributions is the foundation for analyzing the historical biogeography of *Pterocladiella* using recently devised analytical tools. Tectonic events, long-distance dispersal, and isolation have produced a complicated mosaic of relic species, geographically separated sister species, and introductions in this genetically diverse and morphologically simple genus. We caution that our estimates of the timing of evolutionary events must be interpreted with care due to the scarcity of red algae in the fossil record with which to calibrate the timeline (Yang et al., 2016; Yoon et al., 2016; Leliaert et al., 2018).

*Pterocladiella* likely arose in the Tethys Sea in the Cretaceous period in a region that now corresponds to the tropical shallow reefs of Eastern Africa, northern Australia, and South/Southeast Asia (Figure 3). Cretaceous Tethys Sea origins have also been inferred in the red seaweed *Portieria* (Leliaert et al., 2018), and the brown seaweed *Lobophora* (Vieira et al., 2017), as well as in several groups of corals, fishes, and other tropical marine animals (e.g., Tittensor et al., 2010; Bowen et al., 2013; Cowman et al., 2017).

Early diversification of the genus may have been driven by vicariance through tectonic events in the Cretaceous (Figure 3; Supplementary Table 4), a time when the Indo-Madagascar subcontinent formed (120–112 Ma) and separated into India and Madagascar (89–83 Ma; Masters et al., 2006). The narrow ranges of the early diverging species, *P. caespitosa*, *P. feldmannii* and *P. hamelii* in South Africa and Madagascar (clade VI), are suggestive of Tethyan relicts in the southwestern margin of the Tethys Sea, consistent with high levels of endemism in marine animals and seaweeds in the southwestern Indian Ocean (Yoder and Nowak, 2006; Warren et al., 2010; Boo et al., 2018).

Long-distance dispersal likely played an important role in the migration of the Central Indo-Pacific ancestors to Northwestern Pacific, Temperate Australia, Eastern Pacific, and Eastern Atlantic during the Cretaceous (Figure 3). Age estimates indicate that the Indo-Pacific species arose during the Eocene to Miocene. The emergence of various shallow-water habitats in the Tethys Sea during these periods likely provided opportunities for diversification and the long-term persistence of species, as has also been suggested for other seaweeds such as *Portieria*, *Lobophora*, and Udoteaceae (Vieira et al., 2017; Leliaert et al., 2018; Lagourgue et al., 2022).

Our analysis estimates that Group V arose in the Central Indo-Pacific and the Eastern Atlantic during the Paleocene. Vicariance events between the Central Indo-Pacific (A) and the Eastern Atlantic (H; Figure 3) long before the closing of the Indian Ocean-Mediterranean Seaway (~13 Ma; Old World Barrier [OWB] in Figure 1D; Bialik et al., 2019) permitted the persistence of phylogenetic connectivity in the Tethys Sea from the Paleocene to the Eocene. The Omani *Pterocladiella* sp15 (clade I) and the Mediterranean *P. melanoidea* (clade V) are likely relics, isolated after the closure of the OWB. This scenario is concordant with the fossil record of marine animals, which suggests past high biodiversity in the northwestern Indian Ocean, with subsequent eastward transfer to marine biodiversity hotspots (Renema et al., 2008; Bowen et al., 2013; Hou and Li, 2018).

The Eastern Pacific species (clade IV) likely originated through a single dispersal event, and remained isolated by the Eastern Pacific Barrier (EPB; Figure 1D), the world’s widest marine biogeographic barrier composed of 5,000 km of deep water (Darwin, 1880; Chow et al., 2011; Baums et al., 2012). The efficacy of the EPB for the isolation and subsequent diversification of the Eastern Pacific clade has yet to be demonstrated in other benthic seaweeds.

The Pacific North American (*P. luxurians* and sp.14) and South American species (*P. andresii*, *P. caloglossoides*, and sp.11) diversified during the Oligocene to Miocene, coinciding with the Central American Seaway (CAS) acting as a barrier, separating northern and southern species, until the Miocene (Cowman and Bellwood, 2013; Hou and Li, 2018). Notably, the presence of both “*Gelidiella calcicola*” and “*Gelidiella feldmannii*” suggests that ancestral species was widespread from the Eastern Pacific to the Atlantic Ocean *via* the CAS (Figure 3; Supplementary Figure 6). Thus, the CAS may have functioned as a biogeographical barrier between North and South America in the Eocene, but it acted as a dispersal pathway for the Atlantic species. It may also have served as a passage for more recent vicariant species between the Central Indo-Pacific and Caribbean Sea.

As reported in other marine animals and seaweeds (Williams and Duda, 2008; Leliaert et al., 2018), early divergence in *Pterocladiella* preceded the barriers separating the Atlantic and Indo-Pacific (EPB, OWB, and IOP); these major geological events do not appear to have affected diversification rates in *Pterocladiella*. Diversification of *Pterocladiella* occurred relatively consistent over ~100 million years from the Early Cretaceous to the present, as has been found in other seaweeds (Vieira et al., 2017; Leliaert et al., 2018; Lagourgue et al., 2022). In contrast, for some groups of corals, fishes, and gastropods, increased diversification rates have been inferred in the Late Cretaceous or in the Oligo-Miocene, likely due to geographical complexity caused by tectonic changes (Williams and Duda, 2008; Leprieur et al., 2016).

### Sister Species: Distribution and Modes of Speciation

The distribution patterns of nine pairs of sister species provide insights into three speciation modes: peripatry, sympatry, and allopatry (Table 2; Supplementary Figure 9). Our proposal on the peripatric speciation is that species pairs occurred in a close distance of the same marine bioregion to move by various modes of dispersals. The asymmetrical pattern of distribution for those pairs and the absence of physical barrier also suggests peripatric speciation. Four sister pairs appear to be peripherally and asymmetrically isolated (peripatry; Supplementary Figures 9A–D). About 21.5 Ma, the divergence of *Pterocladiella luxurians* (southern California) and sp.14 (Monterey to British Columbia) may be the result of a directional shift along a strong thermal gradient in the Pacific North Ameri*ca. Pterocladiella andresii* from Coquimbo, Chile, occurs about 650 km from Robinson Crusoe Island, where its sister sp.11 occurs. Their divergence likely occurred in the Pliocene (~4.7 Ma), simultaneously with the volcanic rise 3.8–4.2 Ma of Robinson Crusoe Island. The Malaysian *P. megasporangia* likely diverged at the western margin of the range of *P. megasporangia2* (Indonesia, Vietnam, and Taiwan) around 4.8 Ma, pre-dating the opening of the Strait of Malacca at the beginning of the Quaternary (ca. 2.6 Ma). Similarly, *P. australafricanensis2* from Madagascar and Oman likely diverged about 5.0 Ma from the widespread *P. australafricanensis*. The peripatric species pairs that we proposed here are rare in red seaweeds and remain an intriguing issue to be verified by further study.

For three pairs, sister species partially or completely overlap in distribution (sympatry; Supplementary Figures 9E–G). *Pterocladiella* sp.5 (2 specimens from Hon Gam Gi) and sp.8 (18 specimens from Hon Gam Gi and two other locations) diverged in the Miocene (~12.5 Ma) but both occur at the same site (Phu Quoc Island, Vietnam). These two species have an asymmetric distribution in Phu Quoc Island, since their divergence in the middle Miocene. *Pterocladiella tenuis* and sp.10, both from the southwestern coast of Korea, diverged during the Pliocene (~4.9 Ma; Boo et al., 2010; this study). The widespread species, *P. caerulescens* diverged from sp.1 in the Miocene (~7.3 Ma); they overlap in New Caledonia (Supplementary Table 4). However, it is difficult to determine whether two sister species have sympatry sorely or sympatry after a first allopatric divergence.

Two sister pairs, *P. beachiae* and *P. beachiae2*, and *P. santarum* and sp9, have been separated by the Isthmus of Panama since their vicariant and allopatric divergences during the Late Miocene (Supplementary Figure 9H). Similarly, Isthmian geminate sisters in marine animals diverged during a long gradual closure of the Isthmus of Panama from 12 to 2.8 Ma (Cowman and Bellwood, 2013; O’Dea et al., 2016). Closely related species are often widely separated geographically (Grossenbacher et al., 2014).

Our study suggests that the predominance of peripatry and sympatry, and the asymmetrical distribution of sister species in *Pterocladiella*, represent the process of budding speciation, a process by which small colonizing populations, either at the margin or within broadly distributed ancestral populations, become isolated due to environmental niche differences and rapidly diverge (Mayr, 1954; Barraclough and Vogler, 2000; Grossenbacher et al., 2014). Divergences likely occurred in the Early Miocene to Pliocene, when continents, oceans, and seawater temperatures in the tropics were similar to those today (Robinson et al., 2008). Budding speciation may be common in sessile organisms which have a low dispersal capacity, and many cases of budding speciation likely have been reported under peripatric speciation (Crawford, 2010; Anacker and Strauss, 2014; Grossenbacher et al., 2014). To better assess what speciation scenarios are at play in *Pterocladiella* species, further studies combining whole genome sequencing and demographic modeling of the divergence process would be required.

### Recent Long-Distance Dispersal Within Cosmopolitan Species

Haplotype networks confirmed long-distance dispersal within the cosmopolitan species (occurring in three realms), *P. bartlettii*, *P. caerulescens*, and *P. capillacea* (Figure 4). This result contrasts with recent studies that have shown that many allegedly widespread red algal species are actually multiple cryptic species with narrow ranges (Won et al., 2009; Díaz-Tapia et al., 2018; Gabrielson et al., 2018; Leliaert et al., 2018; but see Calderon et al., 2021). For *P. bartlettii*, there was a close relationship between individuals from Vietnam and Brazil (~17,000 km apart), indicating recent long-distance dispersal or introductions, though we cannot discern the source vs. sink. Madagascan populations, linked to BAR3 by five missing haplotypes, likely represent a relict population. *Pterocladiella caerulescens*, with a range of ~18,000 km (Hawai’i to Madagascar), shows a high diversity in Southeast Asia, but those populations are distantly related to the haplotypes from Sri Lanka, New Caledonia, Madagascar and Western Australia. This geographic structure may have been generated by the Sunda Shelf Barrier (SSB, Figure 1D), as reported in fish (Bay et al., 2004; Lourie and Vincent, 2004). The presence of an identical haplotype in China (Hainan Island) and Hawai’i suggests a recent introduction. *Pterocladiella capillacea*, with a range of ~18,000 km (Brazil to Korea), revealed more complex pattern, with varying degrees of connection among areas, and strong isolation of populations from Brazil, Hawai’i, Indonesia, and eastern Japan. The close relationship between the Mediterranean and Asian haplotypes may be a result of a recent range-expansion and/or introduction. Our result supports that the widespread species have experienced sufficient amount of gene flow to maintain species cohesion in haplotype analysis (Figure 4). This is supported by previous studies on those species from Brazil, China, and Madagascar (Boo et al., 2016b; Iha et al., 2017; Wang et al., 2020).

The Central Indo-Pacific was the most likely area of origin for both *P. bartlettii* and *P. caerulescens*; they subsequently spread *via* long-distance dispersal to the Western Indo-Pacific, the Eastern Indo-Pacific and the Western Atlantic (Figure 3). Similarly, *P. capillacea*, which originated in the northwestern Pacific, likely spread to the Atlantic, Australasia and Indo-Pacific regions. Additional population-level sampling and the use of various nuclear markers (e.g., using whole genome sequencing) will be needed to evaluate long-distance dispersal scenarios and phylogeographic patterns in these widespread species.

Long-distance dispersal events across biogeographical barriers are challenging to explain for *Pterocladiella* species having low-dispersal capacity. A possible explanation of short to long distance dispersal includes dispersal through currents within similar thermal zones (Lüning, 1990; Fraser et al., 2013), where seamounts may function as stepping-stones. Long distance dispersal may also occur through rafting by volcanic pumice, tsunami debris, tar lumps; hitchhiking attached to boat and ship and ballast water may cause the dispersal of non-buoyant seaweeds (van den Hoek, 1987; Norton, 1992; Winston, 2012; Hansen et al., 2018). *Pterocladiella* tissues have abundant rhizoidal filaments of cellulose bundle, typical of the Gelidiales but not in other seaweeds (Felicini and Perrone, 1994; Boo et al., 2016b), which may be slow in digestion compared to other red seaweeds. It may be possible that undigested thalli in the stomach of green turtles and fishes (Awabdi et al., 2013; Vermeij et al., 2013), as well as eroded stipes or holdfasts on mollusk shells (see Figure 1C) may survive in remote locations, as reported in the red alga *Centroceras* (Ceramiales; Lipkin, 1977). Vermeij et al. (2013) reported the Gelidiales species were highly capable of growth after gut passage and suggested that the red seaweeds can use animal vectors for dispersal. Re-attachment capacity likely contributes to the success of sporadic dispersal events. *Pterocladiella* species often produce new branches from damaged tissues or new plants from excised branches (Felicini and Perrone, 1994). This vegetative reproduction may be key to population persistence, after long-distance transport or human-mediated introductions of *Pterocladiella*. Further studies are needed to decipher geographical structure and introduction routes of widespread species.

## Conclusion

Our study is a major step toward clarifying the diversity and biogeographic history of the widely distributed red algal genus *Pterocladiella*. Despite low dispersal capacity spores, the genus has attained a global distribution and diversified into 43 species since its estimated origin in the Tethys Sea during the Early Cretaceous. The monophyly of Eastern Pacific species demonstrates the role of the Eastern Pacific Barrier, an intriguing issue to be verified in other red seaweeds. Divergences between sister species from the Central Indo-Pacific and the Caribbean Sea likely occurred by vicariance events. Asymmetrical distributions and predominance of peripatry and sympatry between sister species highlights the importance of budding speciation in *Pterocladiella*, which is probably common in sessile organisms (Crawford, 2010; Anacker and Strauss, 2014). The persistence of congeneric groups, sister species, and cosmopolitan species across remote regions also suggests the likelihood of long-distance dispersal. Because dispersal is typically extremely short in sessile marine plants and seaweeds (Kinlan and Gaines, 2003; Kerswell, 2006), animal vectors or vegetative branches of *Pterocladiella* might function as diaspores for long-distance dispersal. A high capacity for regeneration in *Pterocladiella* (Felicini and Perrone, 1994) might have increased their colonization success in remote locations. *Pterocladiella* species at the deep nodes may have arisen from vicariance processes, associated to the open of Tethys Sea, mixed processes of vicariance and dispersal after the closure of Tethys, and recent, cross-ocean dispersal. Further phylogeographic and population genetics approaches will demonstrate either natural long-distance dispersal and/or anthropogenic has shaped widespread distribution. Our results are congruent with biogeographical patterns in other seaweeds, but the origin and subsequent diversification of the Eastern Pacific clade is unique. Our detailed investigation of this genus contributes to the methodology and conceptualization of historical biogeography for other widely dispersed organisms with low-dispersal capacity.

## Data Availability Statement

The datasets presented in this study can be found in online repositories. The names of the repository/repositories and accession number(s) can be found in the article/Supplementary Material.

## Author Contributions

GHB and HSY conceived and designed the study. GHB, LLG, EC, ODC, TVN, CP, and KAM performed the collections and provided samples. GHB and FL analyzed and interpreted the data. HSY supervised the research. All authors contributed to the article and approved the submitted version.

## Funding

This study was supported by Basic Science Research Program through the National Research Foundation of Korea (NRF) by the Ministry of Education (2018R1A6A3A03012648 and 2021R1I1A1A01049542), postdoctoral funding from the Silva Center for Phycological Documentation, University Herbarium, University of California at Berkeley, and the European Marine Biological Resource Centre (EMBRC)-France, whose French state funds are managed by the ANR within the “Investing for the future program” under reference ANR-10-INBS-02 to GHB; LLG acknowledges support for the organization of expeditions from Fonds Européen de Développement Régional (FEDER) and Port Autonome de la Guadeloupe, Total Foundation, Prince Albert II of Monaco Foundation, Fondation EDF, Stavros Niarchos Foundation and Entrepose Contracting, in-kind support from the Divine Word University (DWU), and post-expedition support from Agence Nationale de la Recherche (ANR) and the National Science Council of Taiwan (ANR TF-DeepEvo 12 ISV7 005 01), European Regional Development Fund (ERDF), the Territorial Collectivity of Martinique (CTM), Plantations Saint-James and BRED, European Regional Development Fund (ERDF/FEDER), Fonds Shell, Région Guyane, Conseil Général de la Guyane, Direction de l’Environnement, de l’Aménagement et du Logement (DEAL), and Direction Régionale de la Recherche et de la Technologie (DRRT); and the Collaborative Genome Program of the Korea Institute of Marine Science and Technology Promotion (KIMST) funded by the Ministry of Oceans and Fisheries (MOF; 20180430) and the National Research Foundation of Korea (NRF-2017R1A2B3001923 and 2022R1A2B5B03002312) to HSY.

## Conflict of Interest

The authors declare that the research was conducted in the absence of any commercial or financial relationships that could be construed as a potential conflict of interest.

## Publisher’s Note

All claims expressed in this article are solely those of the authors and do not necessarily represent those of their affiliated organizations, or those of the publisher, the editors and the reviewers. Any product that may be evaluated in this article, or claim that may be made by its manufacturer, is not guaranteed or endorsed by the publisher.

## Footnotes

^1^ http://species.h-its.org/ptp/

^2^ https://bioinfo.mnhn.fr/abi/public/abgd/abgdweb.html
