# Supplementary material for: Ancient Tethyan Vicariance and Long-Distance Dispersal Drive Global Diversification and Cryptic Speciation in the Red Seaweed Pterocladiella
Source: Front Plant Sci. 2022 Jun 2;13:849476. doi: 10.3389/fpls.2022.849476 (PMC9201827; doi:10.3389/fpls.2022.849476)
Supplement: Supplementary file 2 [file Data_Sheet_1.docx]

Supplementary Material


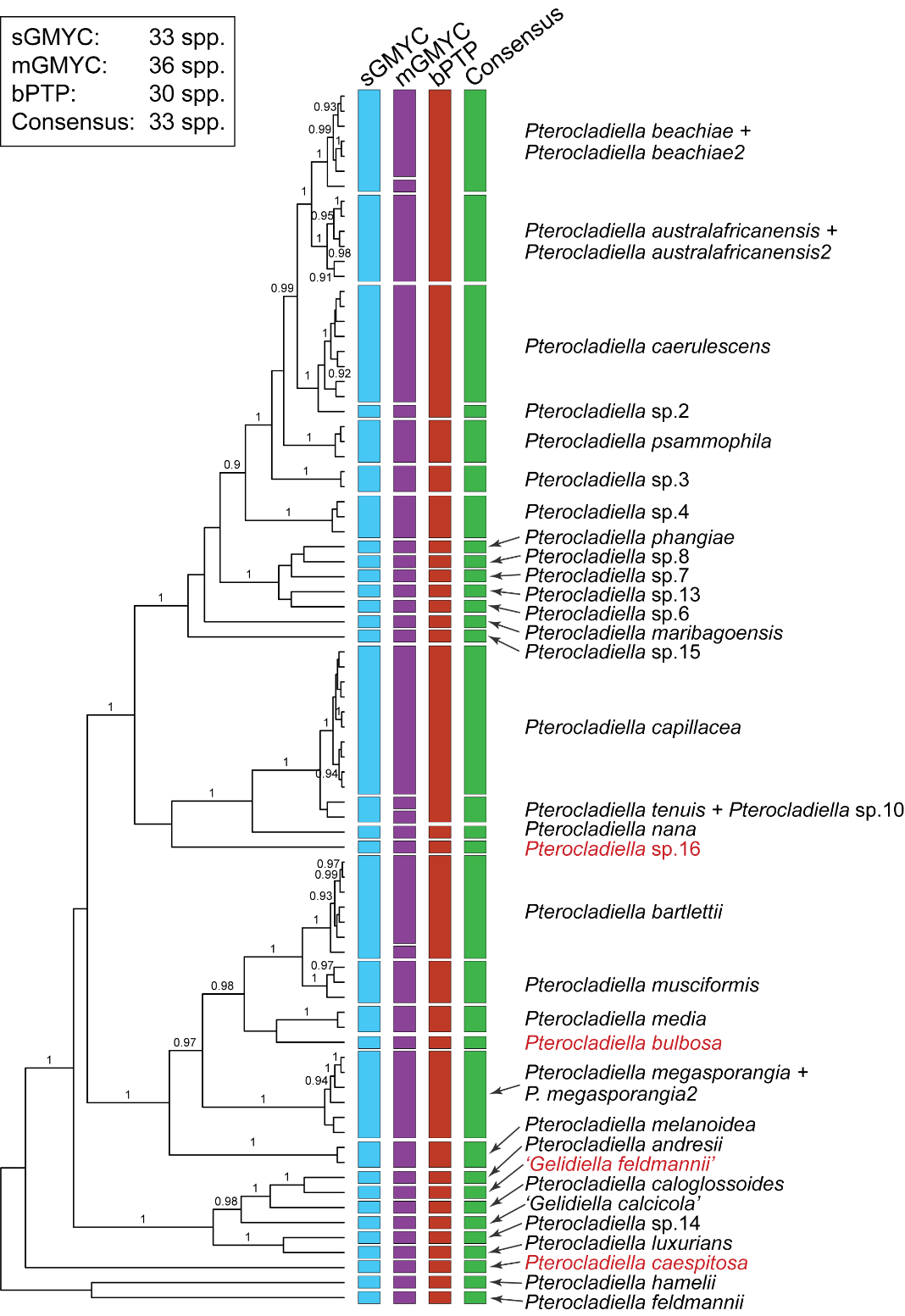


**Supplementary Figure 1.** Results of the three species delimitation methods based on the *rbc*L dataset. Bars represent results from the GMYC method using a single threshold (sGMYC), GMYC with multiple thresholds (mGMYC), and Bayesian poison tree processes method (bPTP). The green bar represents the consensus species delimitation using a conservative approach that matches at least 2 out of 3 species delimitation analyses. Posterior probability is indicated on the nodes.


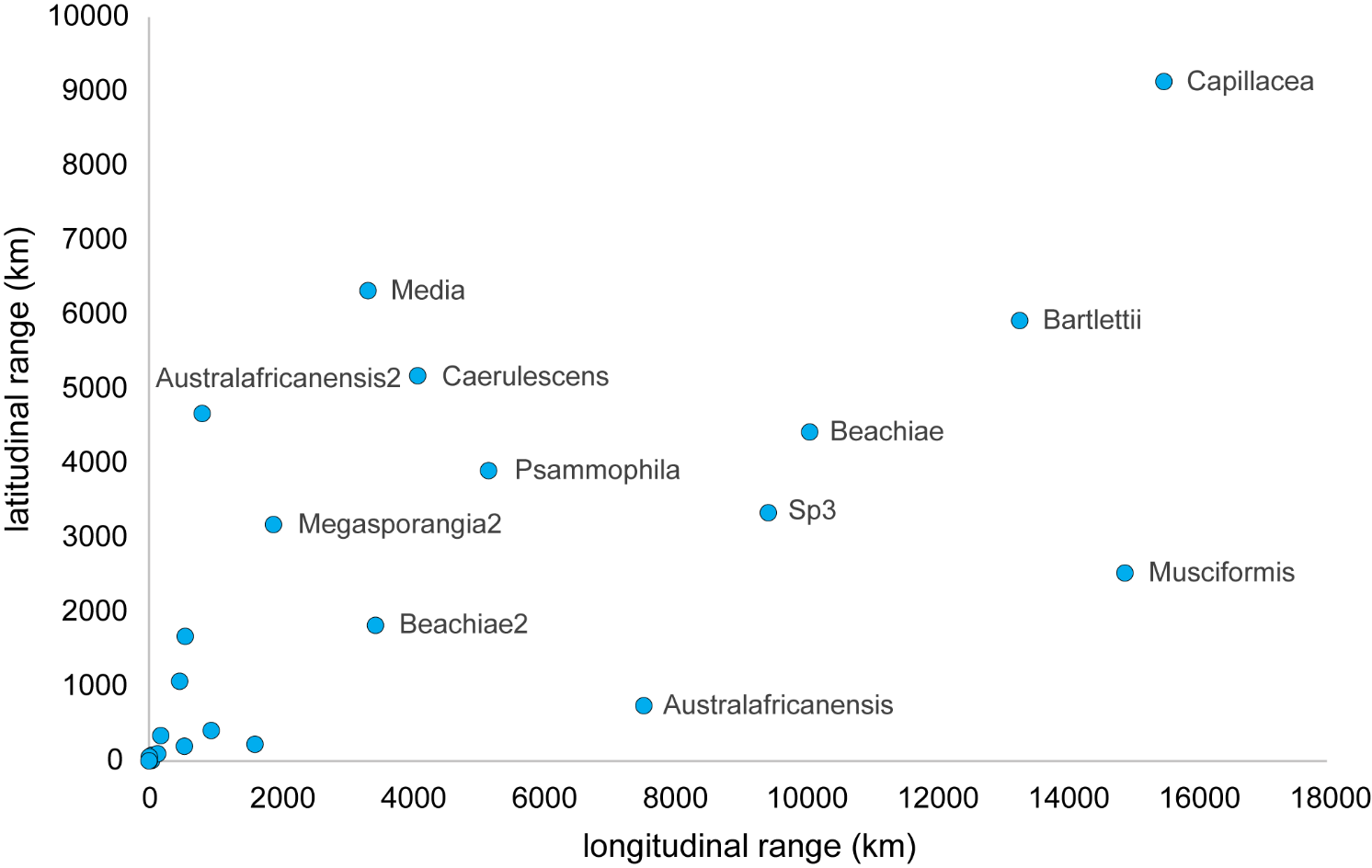


**Supplementary Figure 2.** Latitudinal and longitudinal ranges of the 43 *Pterocladiella* species. Dot indicates geographical region of the species.


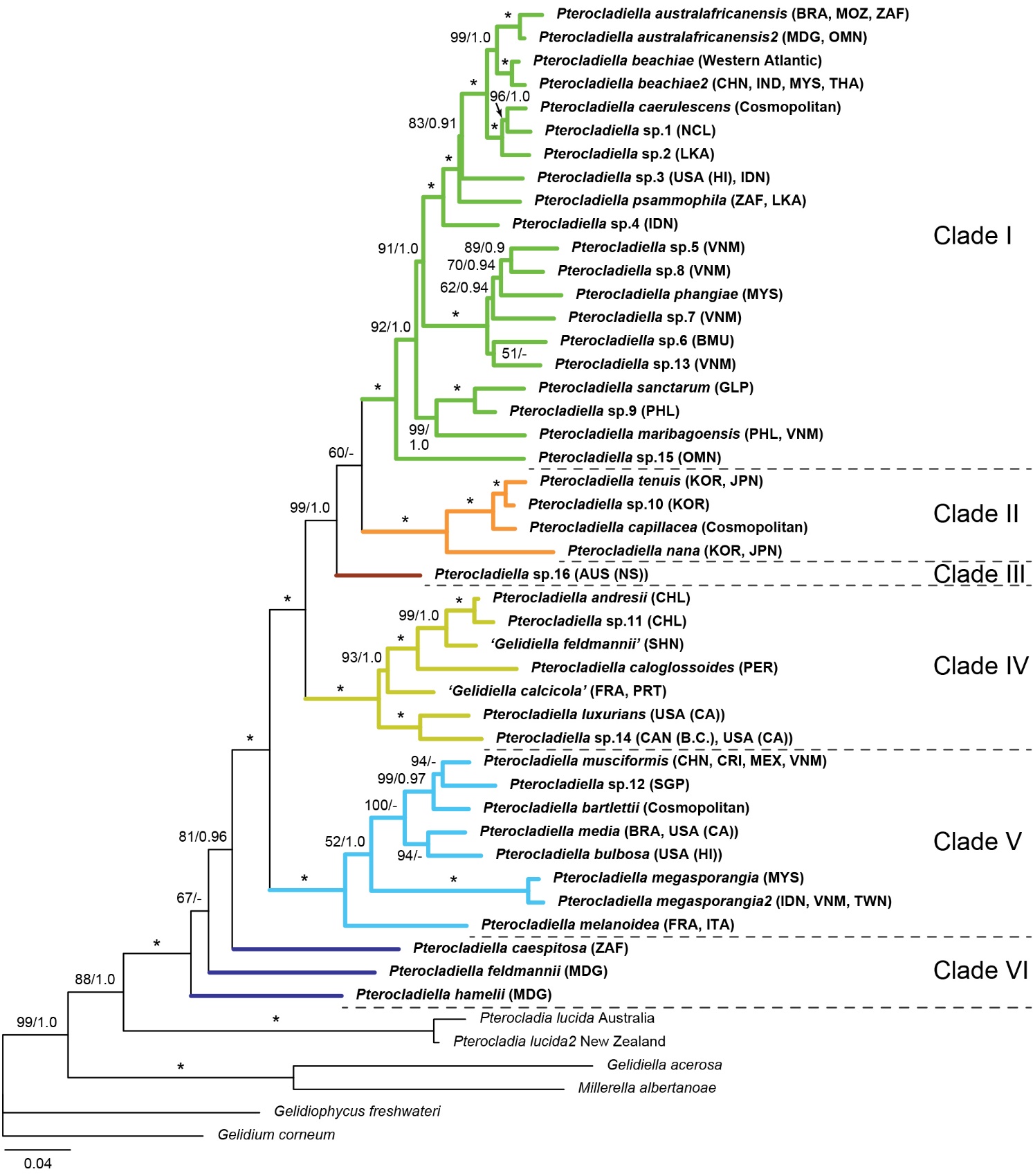


**Supplementary Figure 3.** Phylogenetic tree of *Pterocladiella* obtained by maximum-likelihood inference of the concatenated *cox*1 + *cob* + *psa*A + *psb*A + *rbc*L dataset (4,502 bp). ML and bootstrap values (≥50%) and Bayesian posterior probabilities (≥0.90) are indicated above and below branches, respectively. Asterisks indicate full support in both analyses.


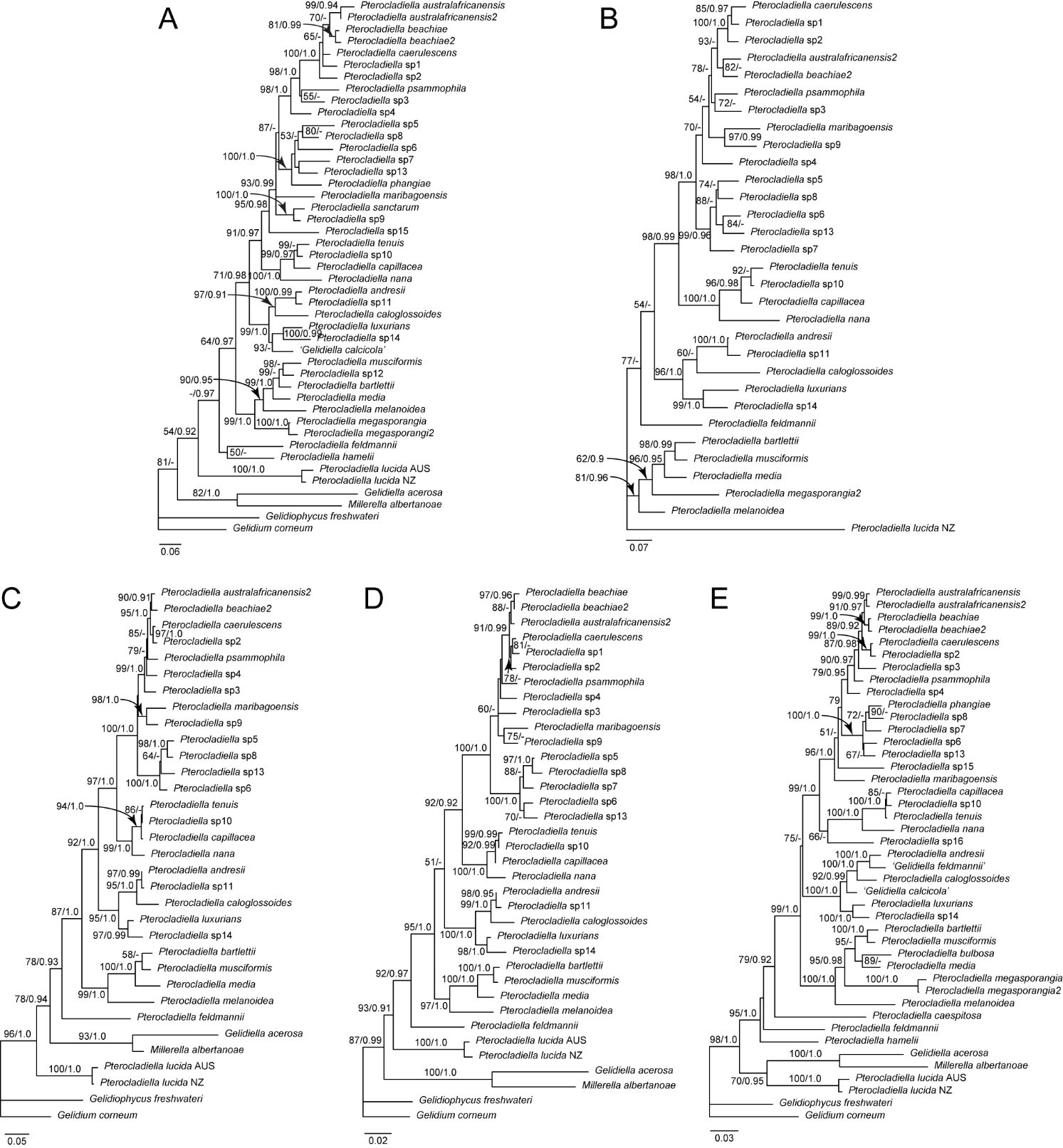


**Supplementary Figure 4.** Maximum likelihood phylogeny of *Pterocladiella* for COI-5P **(A)**, *cob* **(B)**, *psa*A **(C)**, *psb*A **(D)**, and *rbc*L **(E)** sequences. Both ML bootstrap values (≥ 50%) and Bayesian posterior probabilities (≥ 0.9) are indicated at branches.


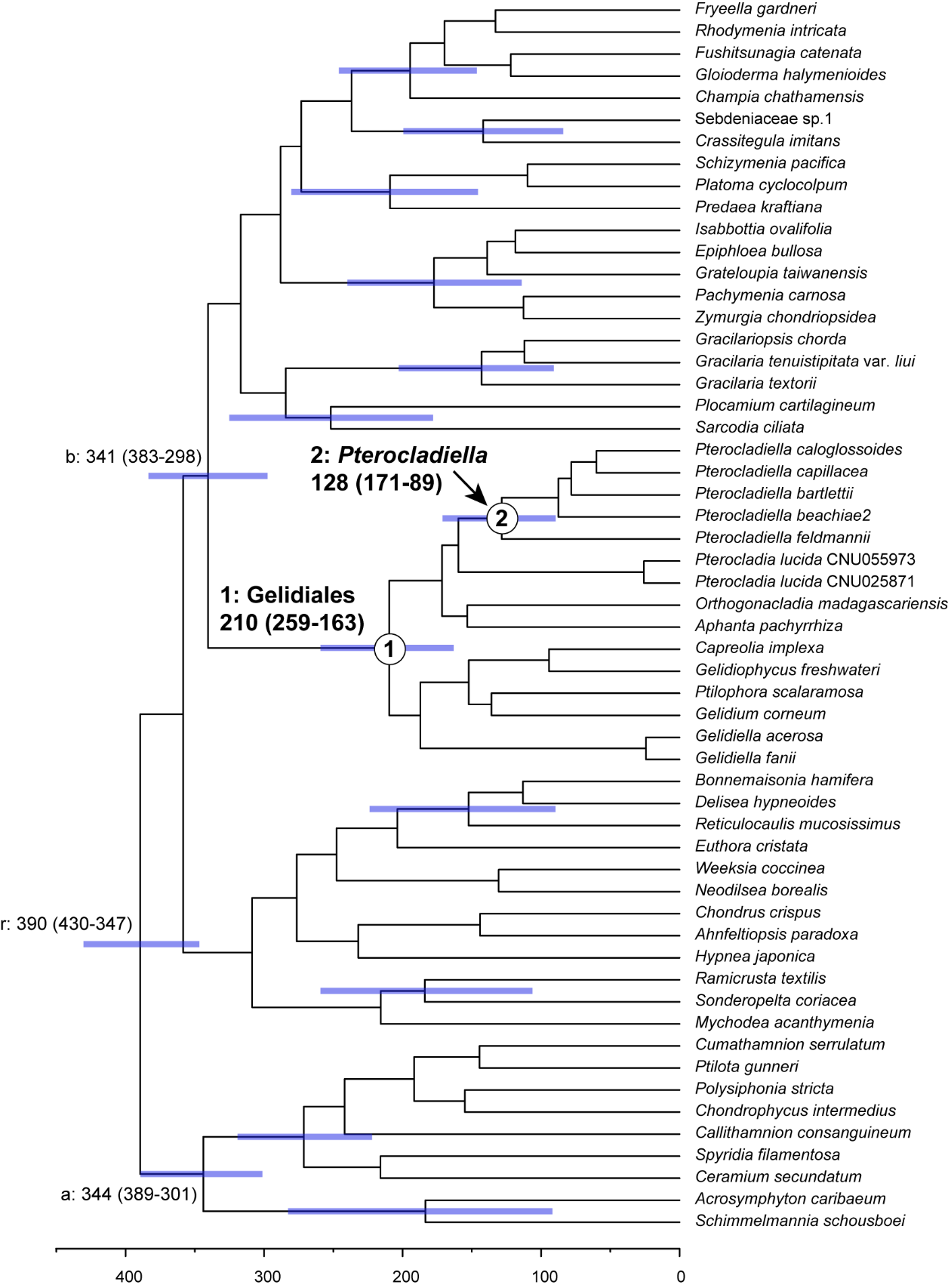


**Supplementary Figure 5.** Chronological dating of the order Gelidiales and crown node of *Pterocladiella* estimated using a Bayesian analysis concatenated data set of four-gene sequences (COI-5P, *psa*A, *psb*A, and *rbc*L). Mean divergence time estimates are presented near nodes and bars represent the highest posterior density (95%).


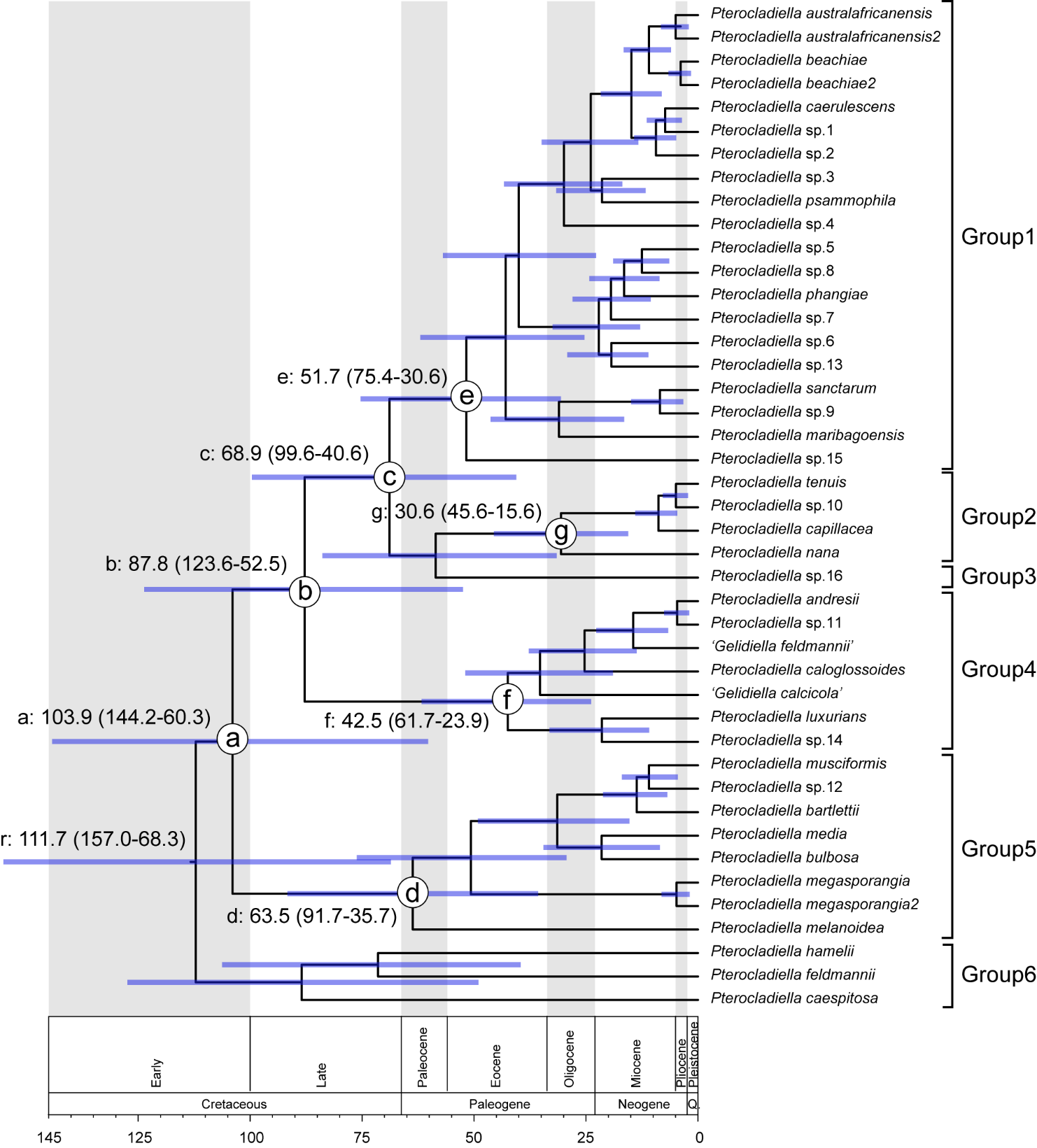


**Supplementary Figure 6.** Chronological dating of species in *Pterocladiella* estimated using a Bayesian analysis concatenated data set of five-gene sequences (COI-5P, *cob*, *psa*A, *psb*A, and *rbc*L). Mean divergence time estimates are presented near nodes and bars represent the highest posterior density (95%).


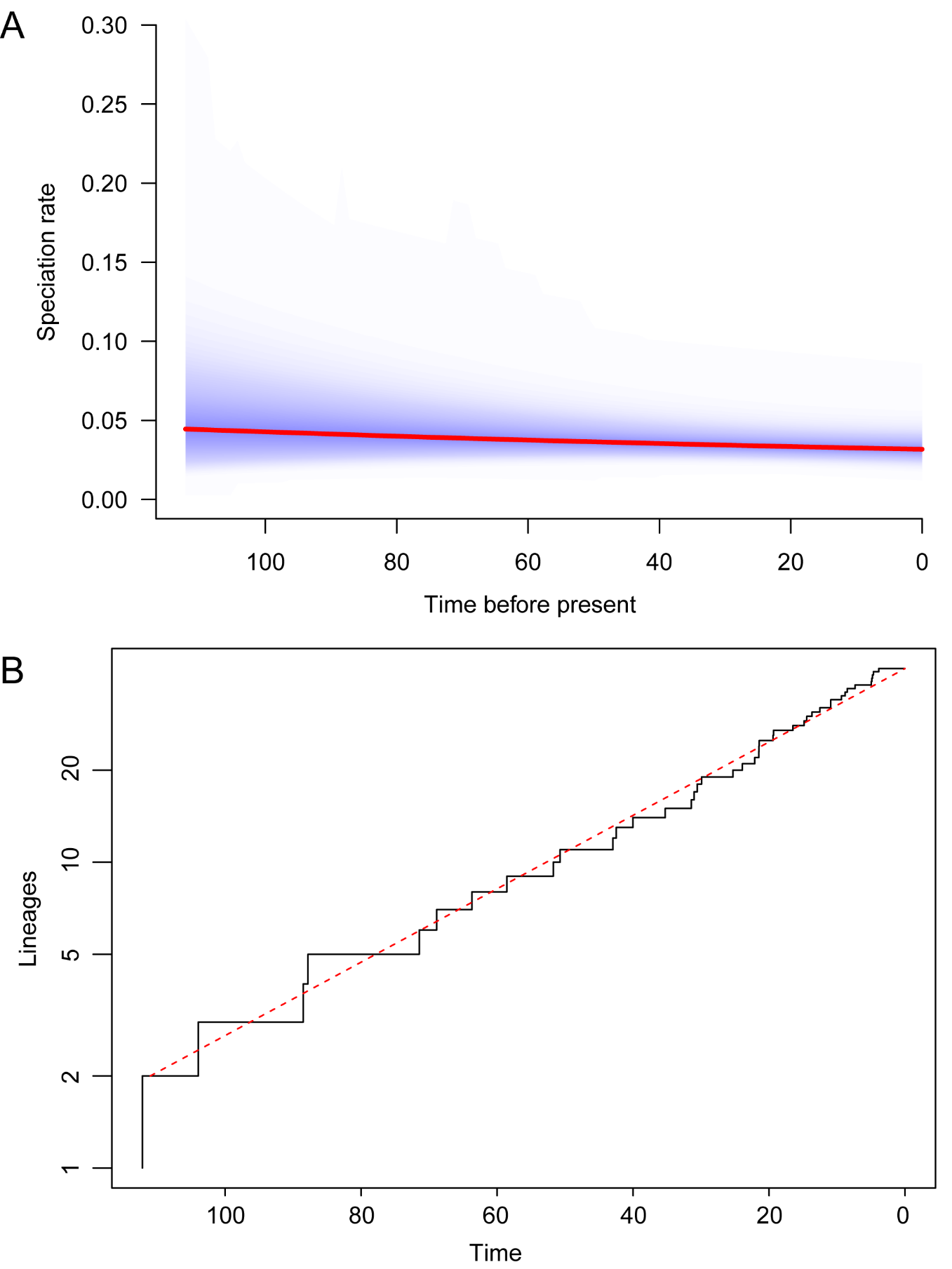


**Supplementary Figure 7.** Speciation rate **(A)** and Lineage-through-time **(B)** plot of *Pterocladiella* derived from the time calibrated phylogeny (Supplementary Fig. S4). The dashed straight line represents a constant rate of diversification with no extinction.


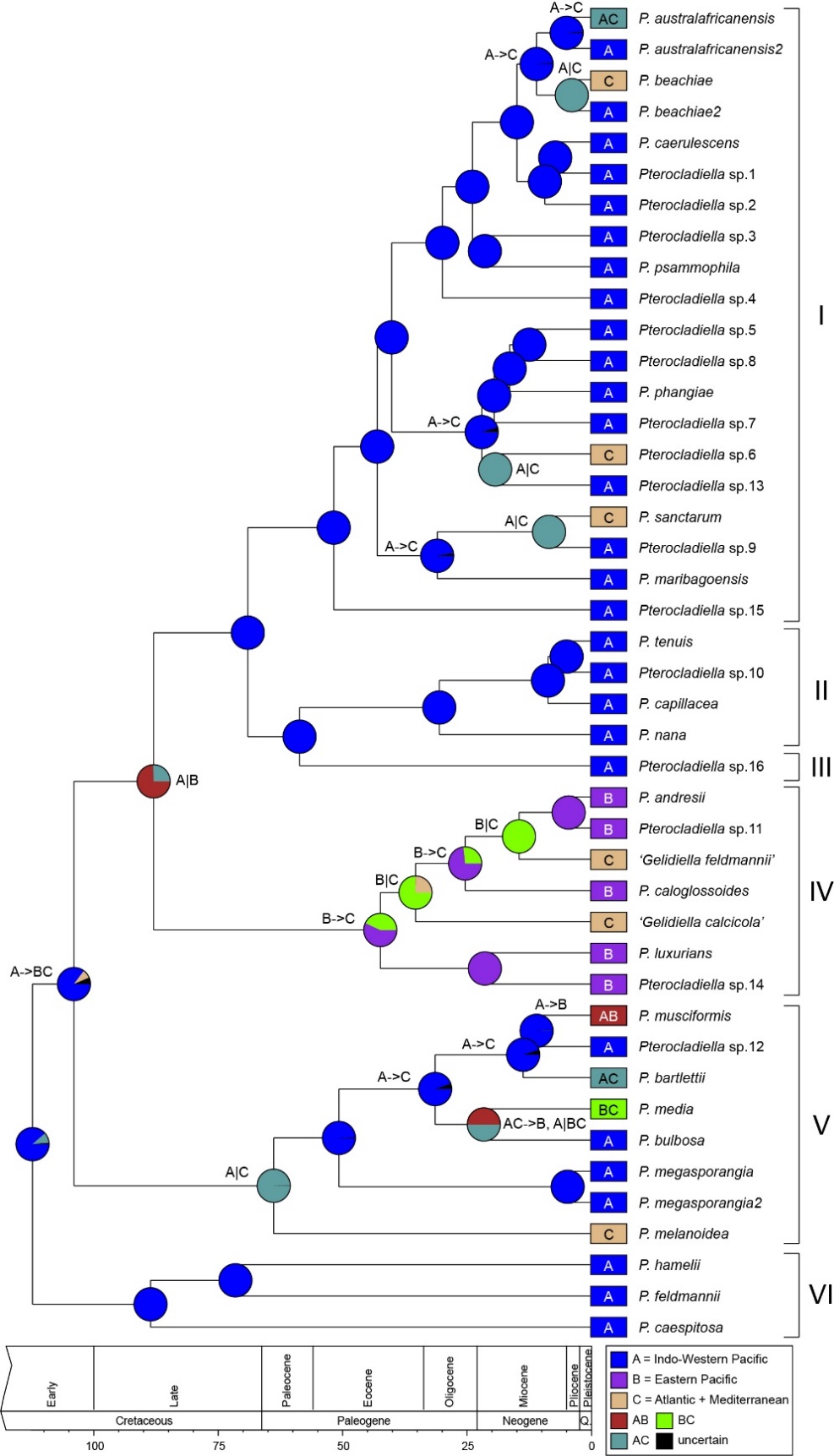


**Supplementary Figure 8.** Ancestral area reconstructions and biogeographical events in the genus *Pterocladiella* under DIVALIKE model considering three geographic regions. Boxes at the tips indicate geographical ranges of extant *Pterocladiella* species. Pie charts represent the probabilities for ancestral area of nodes. The map shows three regions used in the analysis. A color key is provided. The inferred biogeographic events are indicated at nodes and branches (e.g., A->B: dispersal and A|B: vicariance).


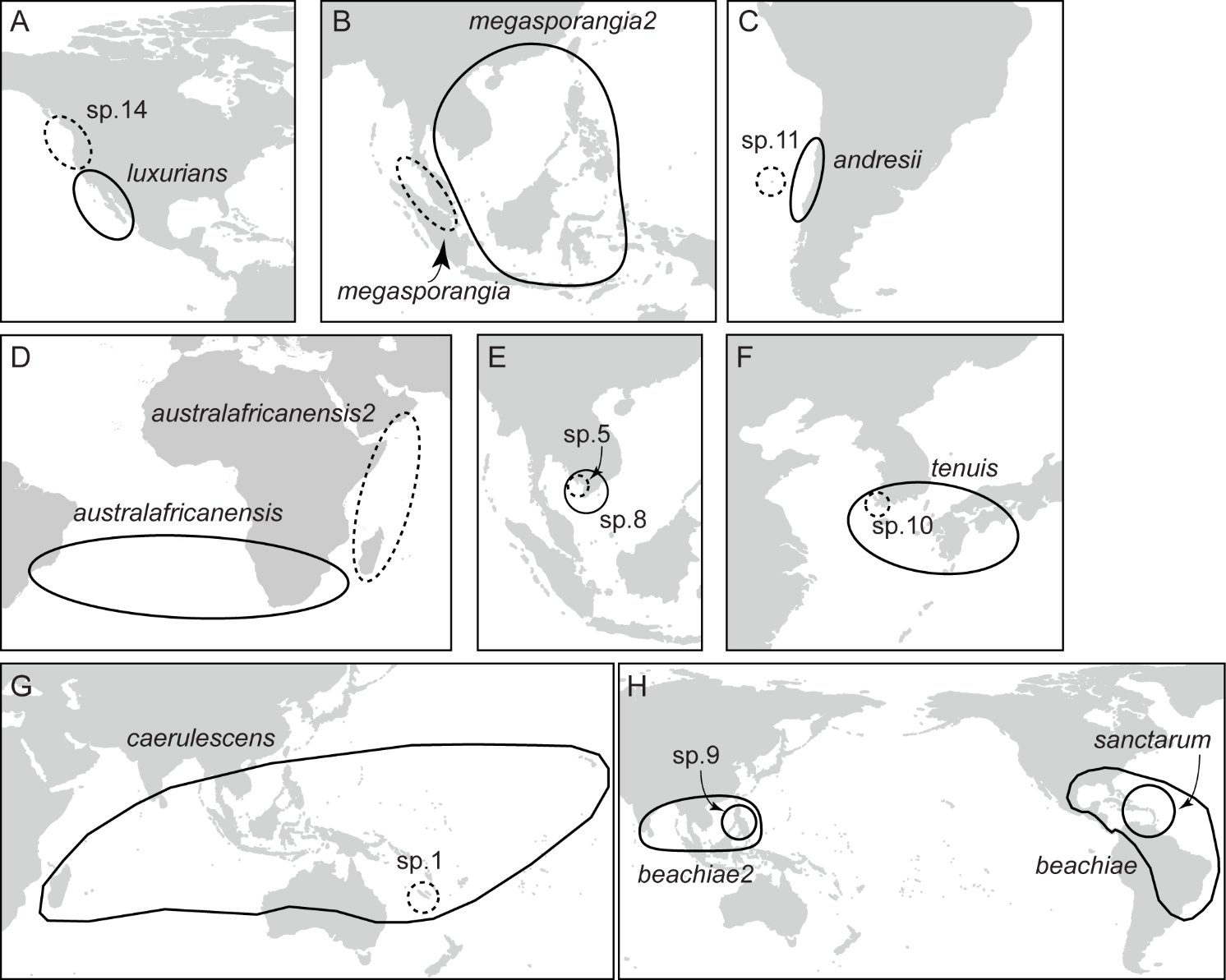


**Supplementary Figure 9.** Maps showing the three distribution patterns of nine pairs of sister species in *Pterocladiella*: peripatry **(A–D)**, sympatry **(E–G)**, allopatry **(H)**. Detailed species’ geographic range is in Supplementary Table S1.

**Supplementary Table 2.** Information of primers used in the present study.

| **Gene** | **Primer name** | **Sequence (5’-3’)** | **References** |
| --- | --- | --- | --- |
| *cox*1 | COXI43F | TCAACAAATCATAAAGATATTGGWACT | Geraldino et al., 2006 |
|  | COXI1549R | AGGCATTTCTTCAAANGTATGATA | Geraldino et al., 2006 |
| *cob* | CB44F | ATTTAATWGATTATCCWACDCC | Saunders and Moore, 2013 |
|  | CB1006R | CAGGCATWCCRCCWATYCAKC | Saunders and Moore, 2013 |
| *psa*A | psaA130F | AACWACWACTTGGATTTGGAA | Yoon et al., 2002 |
|  | psaA971F | ACTACWTCATGGCAYGCWCAACT | Yang and Boo, 2004 |
|  | psaA1110R | CCWATCCACATRTGATGTGT | Yang and Boo, 2004 |
|  | psaA1760R | CCTCTWCCWGGWCCATCRCAWGG | Yoon et al., 2002 |
| *psb*A | psbA-F | ATGACTGCTACTTTAGAAAGACG | Yoon et al., 2002 |
|  | psbA-R2 | TCATGCATWACTTCCATACCTA | Yoon et al., 2002 |
| *rbc*L | F7^*^ | AACTCTGTAGAACGNACAAG | Gavio and Fredericq, 2002 |
|  | F577^**^ | GTATATGAAGGTCTAAAAGGTGG | Freshwater and Rueness, 1994 |
|  | F645^*^ | ATGCGTTGGAAAGAAAGATTCT | Lin et al., 2001 |
|  | F753-Gelidium^**^ | GGAAGATATGTATGAAAGAGC | Boo et al., 2015 |
|  | F1237^**^ | CCAGATGGTATTCAAGCAGGTGC | Freshwater and Rueness, 1994 |
|  | R753^*,**^ | GCTCTTTCATACATATCTTCC | Freshwater and Rueness, 1994 |
|  | R900-Gelidium^**^ | GCGAGAGTATGTTGAATTACC | Boo et al., 2015 |
|  | R1381^**^ | ATCTTTCCATAGATCTAAAGC | Freshwater and Rueness, 1994 |
|  | RrbcS start^*^ | GTTCTTTGTGTTAATCTCAC | Freshwater and Rueness, 1994 |

**rbcL primers for fresh materials, **rbcL primers for type and archival herbarium specimens.*

**Supplementary Table 3.** Summary of results of the four species delimitation methods for mitochondrial COI-5P and plastid *rbc*L sequences.

| **Genes** | **Methods** | **Number of species (confidence interval)** | **Notes** |
| --- | --- | --- | --- |
| Mitochondrial COI-5P | GMYC  singlfe threshold | 44 (38-49) | GMYC model significantly higher than that of the null model of uniform (coalescent) branching rates (L0 = 877.9, LGMYC = 888.0, LR = 20.1, p =4.2e-05***) |
|  | GMYC  multiple threshold | 42 (34-59) | GMYC model significantly higher than that of the null model of uniform (coalescent) branching rates (L0 = 877.9, LGMYC = 889.5, LR = 23.2, p =9.3e-06***) |
|  | bPTP | 38 | Most supported partition found by simple heuristic search |
|  | ABGD | 39 | Distance K2P with MinSlope = 1.3 |
| Plastid *rbc*L | GMYC  single threshold | 33 (30-39) | GMYC model significantly higher than that of the null model of uniform (coalescent) branching rates (L0 = 541.8, LGMYC = 552.4, LR = 21.2, p =2.5e-05***) |
|  | GMYC  multiple threshold | 36 (36-39) | GMYC model significantly higher than that of the null model of uniform (coalescent) branching rates (L0 = 541.8, LGMYC = 553.7, LR = 23.8, p =6.8e-06***) |
|  | bPTP | 30 | Most supported partition found by simple heuristic search |

**Supplementary Table 4.** List of species, type locality, sample used in molecular identification, and distribution pattern of *Pterocladiella* species.

| Species name | Type locality | Sample used in molecular identification | Geographical range confirmed by DNA sequences | Distribution pattern: cosmopolitan, endemic, subcosmopolitan (including disjunct) | References on molecular and/or morphological studies |
| --- | --- | --- | --- | --- | --- |
| *Pterocladiella andresii* G.H.Boo, Calderón & S.M.Boo 2017 | Chungungo, Coquimbo, Chile | Holotype, CNU012838 (*S.M. Boo*), 27 Oct. 2011 | Chile | Endemic | Boo et al., 2017; This study |
| *Pterocladiella australafricanensis* E.M.Tronchin & D.W.Freshwater in Freshwater et al. 2010 | Four Buoy Reef, Sodwana Bay, KwaZulu-Natal, South Africa | Topotype, SA04-045 (*W.D. Freshwater*), 19 Apr. 2004 | Brazil, Mozambique, South Africa | Subcosmopolitan (Disjunct): Western Indo-Pacific and Western Atlantic | Tronchin and Freshwater, 2010; Iha et al., 2017 |
| *Pterocladiella australafricanensis2* | – | Specimens from Madagascar and Oman | Madagascar, Oman | Endemic (broad-range): Western Indo-Pacific | Boo et al., 2016; This study |
| *Pterocladiella bartlettii* (W.R.Taylor) Santelices 1998 | Saint Louis du Sud, Haiti | Many specimens from Caribbean Sea, where the type locality is located | Brazil, Costa Rica, Guadeloupe, Guyane Française, Madagascar, Malaysia, New Caledonia, Singapore, USA (Texas), Vietnam | Cosmopolitan: Western Atlantic, Central Indo-Pacific, and Western Indo-Pacific | Thomas and Freshwater, 2001; Sohrabipour et al., 2013; Boo et al., 2016; Iha et al., 2017; This study |
| *Pterocladiella beachiae* Freshwater in Thomas and Freshwater 2001 | Cahuita, Limón, Costa Rica | Holotype, MICH1306658 (*D.W. Freshwater & D.T. Thomas*), 24 Mar. 1999 | Brazil, Costa Rica, Guadeloupe, Martinique, Panama | Endemic (broad-range): Western Atlantic | Thomas and Freshwater, 2001;  Iha et al., 2017; This study |
| *Pterocladiella beachiae*2 | – | Specimens from Southeast Asia | China, India, Malaysia, Thailand | Subcosmopolitan: Central Indo-Pacific and Western Indo-Pacific | Sohrabipour et al., 2013; Boo et al., 2016; Wang et al., 2020; This study |
| *Pterocladiella bulbosa* (N.H.Loomis) Santelices 1997 | Waialau, Molokai, Hawaii, USA | Holotype, UC1884014 (*M. Reed* *338a*), 19 Jul. 1905 | USA (Hawai’i) | Endemic | Loomis, 1960; Santelices, 1997; This study |
| *Pterocladiella caerulescens* (Kützing) Santelices & Hommersand 1997 | Wagap, New Caledonia | Holotype, L0056117 (*L 941.11.91, Vieillard #2103*), 1863; Isotype, L0056116 (*L 941.11.98*) | Australia (WA), China, Madagascar, Malaysia, New Caledonia, Philippines, Singapore, Sri Lanka, USA (Hawai’i), Vietnam | Cosmopolitan: Central, Western and Eastern Indo-Pacific | Santelices and Hommersand, 1997; Millar and Prud’homme van Reine, 2005; Sohrabipour et al., 2013; Boo et al., 2016; Wang et al., 2020; This study |
| *Pterocladiella caespitosa* (Kylin) Santelices 1998 | Isipingo Beach, near Durban, South Africa | Specimen from Treasure Beach, near type locality | South Africa | Endemic | Norris, 1987; Tronchin and Freshwater, 2007 |
| *Pterocladiella caloglossoides* (M.Howe) Santelices 1998 | Isla San Lorenzo, Peru | Specimens from Yacila, Peru; Type lost | Peru | Endemic | Boo et al., 2017; This study |
| *Pterocladiella capillacea*  (S.G.Gmelin) Santelices & Hommersand 1997 | Mediterranean Sea | Specimens from Mediterranean Sea | Australia, Brazil, China, France, Indonesia, Japan, Korea, Galápagos Islands, Mexico, Taiwan, Tunisia, USA (Hawai’i) | Cosmopolitan: Pacific, Atlantic, and Mediterranean Sea | Shimada et al., 2000; Boo et al., 2010; Iha et al., 2017; Manghisi et al., 2019; Wang et al. 2020; This study |
| *Pterocladiella feldmannii* G.H.Boo, L.Le Gall, I.K.Hwang & S.M.Boo 2016 | Nord-Est du phare d’Evatra, Madagascar | Holotype, PC0166269 (*MAD0302*), 10 May 2010 | Madagascar | Endemic | Boo et al., 2016; This study |
| *Pterocladiella hamelii* G.H.Boo, L.Le Gall, I.K.Hwang & S.M.Boo 2016 | Lavanono, Madagascar | Holotype, PC0171542 (*MAD1297*), 8 Jun. 2010 | Madagascar | Endemic | Boo et al., 2016 |
| *Pterocladiella luxurians* (Collins) Boo & Miller in Boo et al. 2016 | Pacific Beach, San Diego County, California, USA | Holotype, UC1878479 (*M. Snyder*), 8 Mar. 1899 | USA (California) | Endemic | Boo et al., 2016; This study |
| *Pterocladiella maribagoensis* G.H.Boo & P.J.L.Geraldino 2016 | Maribago, Cebu, Philippines | Holotype, CNU040562 (*G.H.Boo & P.J.L. Geraldino*), 23 Apr. 2013 | Philippines, Vietnam | Endemic | Boo et al., 2016; This study |
| *Pterocladiella media* (E.Y.Dawson) G.H.Boo & K.A.Miller in Boo et al. 2016 | Neptune Place, La Jolla, California, USA | Isotype, UC1884019 (*Dawson 15609*), 1 Dec. 1956 | Brazil, USA (California) | Subcosmopolitan (Disjunct): Eastern Pacific and Western Atlantic | Boo et al., 2016; Iha et al., 2017; This study |
| *Pterocladiella megasporangia* J.Sohrabipour, P.-E.Lim & C.A.Maggs in Sohrabipour et al. 2013 | Teluk Kemang, Port Dickson, Negri Sembilan, Malaysia | Holotype, KLU PSM12599-1 (*J. Sohrabipour*), 29 Aug. 2011 | Malaysia | Endemic | Sohrabipour et al., 2013 |
| *Pterocladiella megasporangia*2 | – | Specimens from Southeast Asia | Indonesia, Taiwan, Vietnam | Endemic | This study, DNA only |
| *Pterocladiella melanoidea*  (Schousboe ex Bornet) Santelices & Hommersand 1997 | Tangier, Morocco | Specimens from Mediterranean Sea | Italy, Spain, southern France | Endemic | Freshwater et al., 1994; Boo et al., 2016; This study |
| *Pterocladiella musciformis* (W.R.Taylor) G.H.Boo & K.A.Miller in Boo et al. 2016 | Golfo Dulce, Costa Rica | Holotype, UC1884021 (*W.R. Taylor 34-601)*, 7. Mar. 1934 | China, Costa Rica, Mexico, Vietnam | Subcosmopolitan (Disjunct): Central Indo-Pacific and Eastern Pacific | Boo et al., 2016; Wang et al., 2020; This study |
| *Pterocladiella nana* (Okamura) Shimada, Horiguchi & Masuda 2000 | Yura-jima, Shimo-Koshiki Island, Koshiki Islands, Kagoshima, Japan | Topotype (#83, Shimada et al. 2000) from Shimo-Koshiki Island, Kagoshima, Japan | Korea, Japan | Endemic | Shimada et al., 2000; Boo et al., 2010; This study |
| *Pterocladiella phangiae*  J.Sohrabipour, P.-E.Lim & C.A.Maggs in Sohrabipour et al. 2013 | Port Dickson, Negeri Sembilan, Malaysia | Holotype, KLU PSM12598-1 (*J. Sohrabipour*), 29 Aug. 2011 | Malaysia | Endemic | Sohrabipour et al., 2013 |
| *Pterocladiella psammophila* Tronchin & Freshwater 2007 | Ribbon Reef, Sodwana, KwaZulu-Natal Province, South Africa | Holotype,BOL KZN2K4-70 (*E. Tronchin*), 19 Apr. 2004 | South Africa, Sri Lanka | Endemic (broad-range): Western Indo-Pacific | Tronchin and Freshwater, 2007; This study |
| *Pterocladiella sanctarum* (Feldmann & Hamel) Santelices 2007 | Îles des Saintes, Guadeloupe, West Indies | Specimens from Guadeloupe: FRA1753 (Sud Port Louis), FRA1926 (Canal des Saintes) | Caribbean Sea | Endemic | Santelices, 2007 |
| *Pterocladiella tenuis* (Okamura) Shimada, Horiguchi & Masuda 2000 | Enoshima, Kanagawa Prefecture, Japan | Topotype (#320, Shimada et al. 2000) from Enoshima, Kanagawa, Japan | Korea, Japan | Endemic | Shimada et al., 2000; Boo et al. 2017; This study |
| *‘Gelidiella calcicola Maggs & Guiry 1988’* | Carraroe, County Galway, Ireland | Specimens from northeastern Atlantic | Portugal, northern France | Endemic | Díaz-Tapia and Barbara, 2015; This study |
| *‘Gelidiella feldmannii Baardseth 1941*’ | Nightingale Island (St. 117) and the Settlement (St. 160), Tristan da Cunha | Topotype from shade pool at East Landing, Nightingale Islands, Tristan da Cunha | Tristan da Cunha | Endemic | Saunders et al., 2019 |
| *Pterocladiella* sp1 | – | Specimens from New Caledonia | New Caledonia | Endemic | This study, DNA only |
| *Pterocladiella* sp2 | – | Specimen from Sri Lanka | Sri Lanka | Endemic (singleton) | This study, DNA only |
| *Pterocladiella* sp3 | – | Specimens from Hawai’i and Indonesia | USA (Hawai’i), Indonesia | Subcosmopolitan: Central Indo-Pacific and Eastern Indo-Pacific | This study, DNA only |
| *Pterocladiella* sp4 | – | Specimens from Indonesia | Indonesia | Endemic | This study, DNA only |
| *Pterocladiella* sp5 | – | Specimens from Vietnam | Vietnam | Endemic | This study, DNA only |
| *Pterocladiella* sp6 | – | Specimens from Bermuda | Bermuda | Endemic | This study, DNA only |
| *Pterocladiella* sp7 | – | Specimen from Vietnam | Vietnam | Endemic (singleton) | This study, DNA only |
| *Pterocladiella* sp8 | – | Specimens from Vietnam | Vietnam | Endemic | This study, DNA only |
| *Pterocladiella* sp9 | – | Specimen from the Philippines | Philippines | Endemic (singleton) | This study, DNA only |
| *Pterocladiella* sp10 | – | Specimens from Korea | Korea | Endemic | This study, DNA only |
| *Pterocladiella* sp11 | – | Specimens from Robinson Crusoe Island, Chile | Chile (Robinson Crusoe Island) | Endemic | This study, DNA only |
| *Pterocladiella* sp12 | – | Specimen from Singapore | Singapore | Endemic (singleton) | This study, DNA only |
| *Pterocladiella* sp13 | – | Specimens from Vietnam | Vietnam | Endemic | This study, DNA only |
| *Pterocladiella* sp14 | – | Specimens from British Columbia, Canada and California, USA, as *P. caloglossoides* | USA (California), Canada (British Columbia) | Endemic (broad-range); Eastern Pacific | Smith, 1944; Renfrew et al.1989; This study |
| *Pterocladiella* sp15 | – | Specimen from Oman | Oman | Endemic (singleton) | This study, DNA only |
| *Pterocladiella* sp16 (as *P. caloglossoides*) | – | Specimen from eastern Australia | Australia | Endemic (singleton) | Millar and Freshwater, 2005 |

*Distribution patterns: cosmopolitan, occurring in multiple realms; endemic, occurring in a realm; subcosmopolitan occurring in two realms; disjunct, a species is considerably separated from each other geographically; singleton, a species based on a single specimen or a single sequence (Lim et al., 2011). Dash (–) indicates not applicable.*

**Supplementary Table 5.** Latitudinal ranges of *Pterocladiella* species.

| Species | Southern hemisphere | | | Northern hemisphere | | |
| --- | --- | --- | --- | --- | --- | --- |
|  | 0-20°S | 21-40°S | 41-60°S | 0-20°N | 21-40°N | 41-60°N |
| *P. andresii* | - | O | - | - | - | - |
| *P. australafricanensis* | - | O | - | - | - | - |
| *P. australafricanensis2* | - | - | - | O | O | - |
| *P. bartlettii* | - | - | - | O | O | - |
| *P. beachiae* | - | - | - | O | O | - |
| *P. beachiae*2 | - | - | - | O | - | - |
| *P. bulbosa* | - | - | - | - | O | - |
| *P. caerulescens* | O | O | - | O | O | - |
| *P. caespitosa* | - | O | - | - | - | - |
| *P. caloglossoides* | O | - | - | - | - | - |
| *P. capillacea* | O | O | - | O | O | - |
| *P. feldmannii* | - | O | - | - | - | - |
| *P. hamelii* | - | O | - | - | - | - |
| *P. luxurians* | - | - | - | - | O | - |
| *P. maribagoensis* | - | - | - | O | - | - |
| *P. media* | - | O | - | - | O | - |
| *P. megasporangia* | - | - | - | O | - | - |
| *P. megasporangia*2 | - | - | - | O | O | - |
| *P. melanoidea* | - | - | - | - | O | O |
| *P. musciformis* | - | - | - | O | O | - |
| *P. nana* | - | - | - | - | O | - |
| *P. phangiae* | - | - | - | O | - | - |
| *P. psammophila* | - | - | - | O | O | - |
| *P. sanctarum* | - | - | - | O | - | - |
| *P. tenuis* | - | - | - | - | O | - |
| *‘Gelidiella calcicola’* | - | - | - | - | - | O |
| *‘Gelidiella feldmannii’* | - | O | - | - | - | - |
| *Pterocladiella* sp1 | O | - | - | - | - | - |
| *Pterocladiella* sp2 | - | - | - | O | - | - |
| *Pterocladiella* sp3 | - | - | - | O | O | - |
| *Pterocladiella* sp4 | O | - | - | - | - | - |
| *Pterocladiella* sp5 | - | - | - | O | - | - |
| *Pterocladiella* sp6 | - | - | - | - | O | - |
| *Pterocladiella* sp7 | - | - | - | O | - | - |
| *Pterocladiella* sp8 | - | - | - | O | - | - |
| *Pterocladiella* sp9 | - | - | - | O | - | - |
| *Pterocladiella* sp10 | - | - | - | - | O | - |
| *Pterocladiella* sp11 | - | O | - | - | - | - |
| *Pterocladiella* sp12 | - | - | - | O | - | - |
| *Pterocladiella* sp13 | - | - | - | O | - | - |
| *Pterocladiella* sp14 | - | - | - | - | O | O |
| *Pterocladiella* sp15 | - | - | - | O | - | - |
| *Pterocladiella* sp16 | - | O | - | - | - | - |
| Total species: 43 | 5 | 11 | 0 | 22 | 18 | 3 |

**Supplementary Table 6.** Comparison of the ancestral area reconstruction models implemented in BioGeoBEARS using RASP: dispersal-extinction-cladogenesis (DEC), dispersal-vicariance analysis (DIVALIKE), and BAYAREALIKE. Bold indicate best fit models.

| Model | 3 regions | | | 8 realms | | |
| --- | --- | --- | --- | --- | --- | --- |
|  | LnL | AICc | AICc_wt | LnL | AICc | AICc_wt |
| DEC | -55.5 | 115.3 | 0.0012 | -116.2 | 236.6 | 4.99E-05 |
| **DIVALIKE** | **-52.5** | **109.3** | **0.024** | **-115.7** | **235.8** | **6.00E-05** |
| BAYAREALIKE | -72.4 | 149.1 | 5.40E-11 | -126.8 | 257.8 | 9.80E-10 |

*LnL: log-likelihood of the model, AICc: corrected Akaike information criterion, AICc_wt: AICc weight.*

**References of the supplementary material**

Freshwater, D. W., and Rueness, J. (1994). Phylogenetic relationships of some European *Gelidium* (Gelidiales, Rhodophyta) species, based on *rbc*L nucleotide sequence analysis. *Phycologia* 33, 187–194.

Gavio, B., and Fredericq, S. (2002). *Grateloupia turuturu* (Halymeniaceae, Rhodophyta) is the correct name of the non-native species in the Atlantic known as *Grateloupia doryphora*. *Eur. J. Phycol.* 37, 349–559.

Geraldino, P. J. L., Yang, E. C., and Boo, S. M. (2006). Morphology and molecular phylogeny of *Hypnea flexicaulis* (Gigartinales, Rhodophyta) from Korea. *Algae* 21, 417–423.

Lin, S. M., Fredericq, S., and Hommersand, M. H. (2001). Systematics of the Delesseriaceae (Ceramiales, Rhodophyta) based on large subunit rDNA and *rbc*L sequences, including the Phycodryoideae, subfam. nov. *J. Phycol.* 37, 881–899.

Saunders, G. W., and Moore, T. E. (2013). Refinements for the amplification and sequencing of red algal DNA barcode and RedToL phylogenetic markers: a summary of current primers, profiles and strategies. *Algae* 28, 31–43.

Yang, E. C., and Boo, S. M. (2004). Evidence for two independent lineages of *Griffithsia* (Ceramiaceae, Rhodophyta) based on plastid protein-coding *psa*A, *psb*A, and *rbc*L gene sequences. *Mol. Phylogenet. Evol.* 31, 680–688.

Yoon, H. S., Hackett, J. D., and Bhattacharya, D. (2002). A single origin of the peridinin- and fucoxanthin-containing plastids in dinoflagellates through tertiary endosymbiosis. *Proc. Natl. Acad. Sci. U.S.A.* 99, 11724–11729.
